# Investigations into the Sarcomeric Protein and Ca^2+^-Regulation Abnormalities Underlying Hypertrophic Cardiomyopathy in Cats (*Felix catus*)

**DOI:** 10.3389/fphys.2017.00348

**Published:** 2017-06-08

**Authors:** Andrew E. Messer, Jasmine Chan, Alex Daley, O'Neal Copeland, Steven B. Marston, David J. Connolly

**Affiliations:** ^1^Myocardial Function, NHLI, Imperial College LondonLondon, United Kingdom; ^2^The Royal Veterinary CollegeHatfield, United Kingdom

**Keywords:** *Felix catus*, Ragdoll cat, hypertrophic cardiomyopathy, cardiac muscle, Ca^2+^ regulation, troponin, phosphorylation, uncoupling/recoupling

## Abstract

Hypertrophic cardiomyopathy (HCM) is the most common single gene inherited cardiomyopathy. In cats (*Felix catus*) HCM is even more prevalent and affects 16% of the outbred population and up to 26% in pedigree breeds such as Maine Coon and Ragdoll. Homozygous *MYBPC3* mutations have been identified in these breeds but the mutations in other cats are unknown. At the clinical and physiological level feline HCM is closely analogous to human HCM but little is known about the primary causative mechanism. Most identified HCM causing mutations are in the genes coding for proteins of the sarcomere. We therefore investigated contractile and regulatory proteins in left ventricular tissue from 25 cats, 18 diagnosed with HCM, including a Ragdoll cat with a homozygous *MYBPC3* R820W, and 7 non-HCM cats in comparison with human HCM (from septal myectomy) and donor heart tissue. Myofibrillar protein expression was normal except that we observed 20–44% MyBP-C haploinsufficiency in 5 of the HCM cats. Troponin extracted from 8 HCM and 5 non-HCM cat hearts was incorporated into thin filaments and studied by *in vitro* motility assay. All HCM cat hearts had a higher (2.06 ± 0.13 fold) Ca^2+^-sensitivity than non-HCM cats and, in all the HCM cats, Ca^2+^-sensitivity was not modulated by troponin I phosphorylation. We were able to restore modulation of Ca^2+^-sensitivity by replacing troponin T with wild-type protein or by adding 100 μM Epigallocatechin 3-gallate (EGCG). These fundamental regulatory characteristics closely mimic those seen in human HCM indicating a common molecular mechanism that is independent of the causative mutation. Thus, the HCM cat is a potentially useful large animal model.

## Introduction

### Feline HCM mutations and incidence

Hypertrophic cardiomyopathy (HCM) is identified in about 1 in 500 people. It is the leading cause of sudden cardiac death in young adults and results in significant disability in survivors. Mutations in at least 14 genes encoding proteins of the cardiac sarcomere (with over 1,400 variants) are associated with HCM. In most cases, HCM is inherited as an autosomal dominant trait with variable penetrance (Maron et al., 2014), although the causative mutation cannot be identified in about half of the cases. This may be explained by either existence of rare sarcomeric or non-sarcomeric genetic variants, the influence of modifier genes or the presence of phenocopies (Maron et al., 2010; Lopes et al., 2015). Alterations in two genes, β-myosin heavy chain and myosin-binding protein C (*MYBPC3*) account for ~75% of cases where an underlying mutation has been identified (Alfares et al., 2015).

HCM has an exceedingly high prevalence in cats, affecting ~16% of the outbred population (Paige et al., 2009; Payne et al., 2015). This prevalence increases in pedigree breeds such as the Maine Coon where it is estimated at 26% (Gundler et al., 2008). A heritable component for HCM, has also been described in Ragdoll, Siberian, Sphynx, American Shorthair, Cornish Rex, Persian, European, British Shorthair, Bengal, Chartreux, and Norwegian Forest cats (Trehiou-Sechi et al., 2012; Longeri et al., 2013; März et al., 2014). Despite this, causative mutations have only been identified in Maine Coon and Ragdoll cats. In both breeds, a homozygous mutation in *MYBPC3* was identified resulting in amino acid substitution—A31P and R820W in Maine Coons and Ragdolls, respectively (Meurs et al., 2005, 2007). The same R820W mutation has been identified in a human family which exhibits an almost identical phenotype to Ragdoll cats with severe left ventricular wall hypertrophy, arrhythmia, congestive heart failure and sudden cardiac death in homozygotes and only mild and very late expression in heterozygous carriers (Ripoll Vera et al., 2010; Borgeat et al., 2014). Despite analysis of candidate genes in a number of other pedigree breeds no other mutations have been identified to date (Meurs et al., 2009). There is also limited information regarding the influence of modifier genes on left ventricular (LV) hypertrophy (Borgeat et al., 2015) or the presence of phenocopies in the feline population.

### Feline HCM characteristics and similarities with human

Feline HCM closely resembles the comparable human condition with respect to many clinical and pathologic features, and therefore represents an important spontaneously occurring animal model (Kittleson et al., 1999; Maron and Fox, 2015). At the clinical level, feline HCM is a heterogeneous disease, both in terms of presentation and outcome.

Many cats present with signs of congestive heart failure, others die suddenly; however, the majority can have long survival times and die of non-cardiac causes (Rush et al., 2002). Prognostic indicators associated with an increased risk of cardiac death are similar to those identified in human patients and included arrhythmia, extreme left ventricular hypertrophy, reduced atrial and ventricular systolic function, regional wall hypokinesis and a restrictive diastolic filling pattern (Payne et al., 2013). That said, inter-species differences are evident, for instance feline HCM more frequently results in progressive heart failure or arterial thromboembolism as the major complication (Borgeat et al., 2014; Maron and Fox, 2015). The incidence of sudden unexpected death associated with feline HCM has not been fully evaluated in cats but may be lower than seen in human patients (Maron and Fox, 2015; Wilkie et al., 2015). In both species, myofiber disarray, intramural coronary artery disease, interstitial and replacement fibrosis are the hallmarks of HCM on histopathology (Liu et al., 1993; Wilkie et al., 2015). Furthermore, in both humans and cats HCM results in significant diastolic dysfunction, however, both species can develop end-stage cardiomyopathy as part of the natural course of disease, where pathologic remodeling includes left ventricular chamber dilation, wall thinning, and fibrosis (Cesta et al., 2005).

### Current studies on function in HCM

Despite progress in elucidating the genetic basis of HCM, there is less understanding of the molecular events that lead from mutation to disease phenotype (Lopes and Elliott, 2014). Development of hypertrophy is a late stage event in HCM, resulting from detrimental processes, which operate during the pre-hypertrophic stage of the disease (Cannon et al., 2015). Studies on septal myectomy tissue from human patients with HCM and from engineered rodent models indicate that the majority of sarcomeric mutations enhance myofilament Ca^2+^-sensitivity leading to a hypercontractile phenotype with energy deficiency and altered Ca^2+^ handling which act as the major common pathways promoting HCM (Huke and Knollmann, 2010; Marston, 2011; Song et al., 2013). Given the striking similarities between the human and feline disease outlined above, it is likely that similar common pathways initiate and drive a significant proportion of the HCM phenotype in cats. Although, contractile and electrophysiological properties of ventricular cardiomyocytes isolated from clinically normal cats and those exposed to pressure loading techniques have been assessed in detail over the last three decades (Kleiman and Houser, 1988; Weisser-Thomas et al., 2005), there remains a sparsity of information concerning the effect of HCM on contractile function in feline cardiomyocytes.

We therefore studied contractile and regulatory proteins in left ventricular tissue from a range of cats with and without evidence of HCM on echocardiography and/or histopathology. These included pure-bred cats such as Ragdoll with and without the homozygous R820W mutation and a number of outbred cats with unknown mutations. Feline samples were compared with myectomy samples from human patients.

We measured protein expression, troponin I and MyBP-C phosphorylation levels and isolated troponin from cat heart muscle and investigated regulation by *in vitro* motility assay.

We found that fundamental Ca^2+^-regulatory abnormalities in cats are virtually the same as in humans indicating this as a potentially useful animal model of hypertrophic cardiomyopathy.

## Methods

### Cat heart collection methods

**Figure 1:**
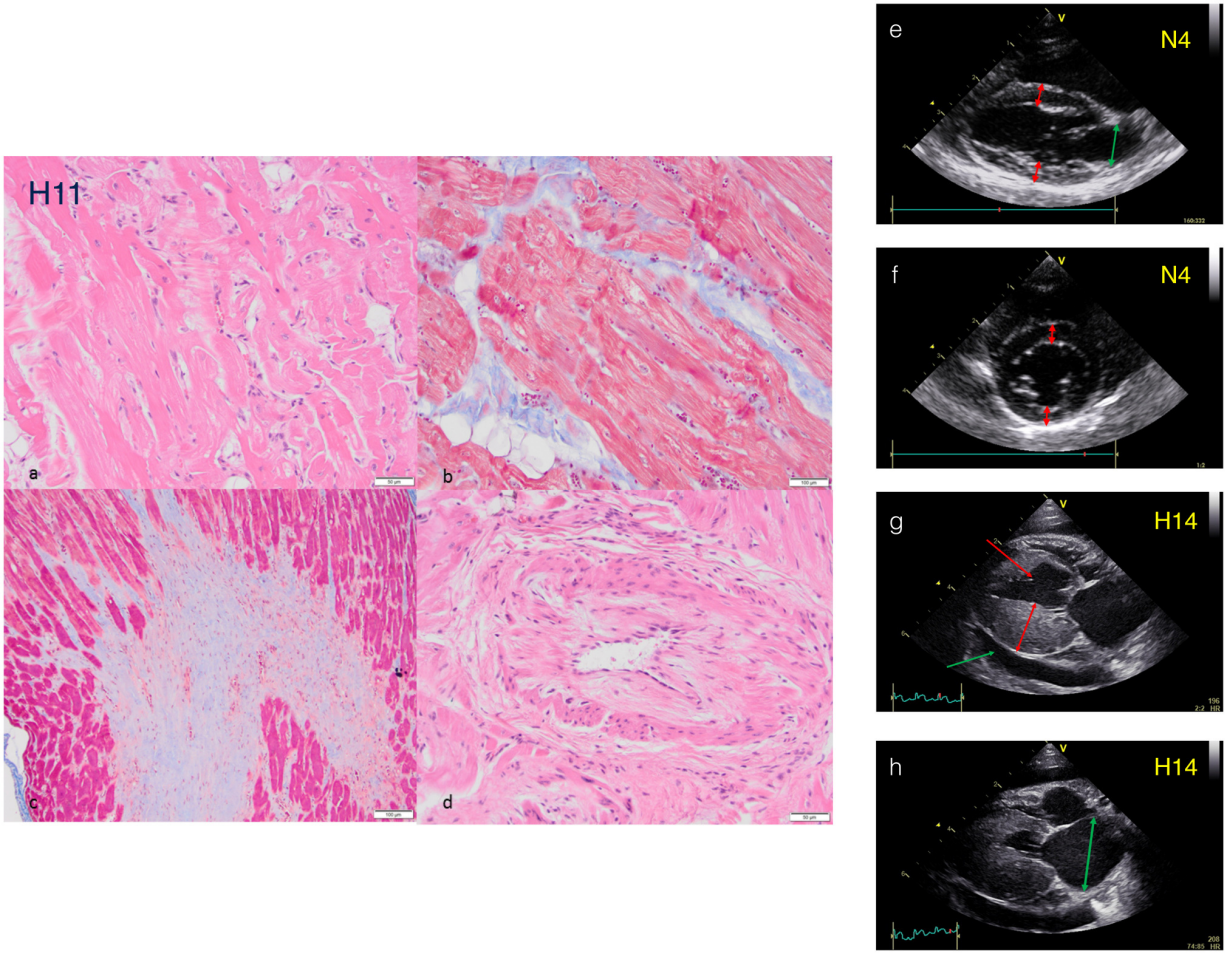
Histopathology and echocardiography characterization of HCM cats. *Histopathology sections from HCM cat H11:* **(a)** Myofibre disarray and sarcoplasmic fragmentation (H+E). **(b)** Intramural fibrosis with myofibre disarray and fragmentation of sarcoplasm (Masson's Trichrome). **(c)** Fibrosis in a papillary muscle (Masson's Trichrome). **(d)** Arteriosclerosis (H+E). *Echocardiography of cat hearts N4 and H14*. **NON-HCM Cat (e)**. Right parasternal long axis view of cat N4 at end diastole. Note normal sized LA (green arrow) and LV wall thickness (red arrows) measuring <6 mm (see accompanying movie S1). **(f)** Right parasternal short axis view at the level of the papillary muscles of cat N4 at end diastole. Note normal LV wall thickness (red arrows) measuring <6 mm. **HCM Cat (g)** Right parasternal long axis view of cat H14 at end diastole. Note severe hypertrophy of the LV free wall measuring >6 mm (double headed arrow) and a small volume pericardial effusion secondary to heart failure (green arrow). An incidental false tendon (single headed red arrow) is also present (see accompanying movie S2). **(h)** Right parasternal long axis view of cat H14 at end systole. Note the severely enlarged LA (double headed arrow). Further echocardiography plots and movies in supplementary Data.

This study was approved by the Royal Veterinary College (RVC) institutional ethics and welfare committee, and written owner consent for study participation was obtained. Full thickness feline myocardium was harvested from the left ventricular (LV) free wall immediately following euthanasia and placed into a pre-cooled tube on dry ice and then transferred within 30 min to a −80°C freezer or stored in liquid nitrogen at below −160°C. Samples obtained from outside the RVC were frozen at −20°C for a maximum of 2 weeks before being transferred to the RVC on dry ice for storage. Cats with HCM were euthanized either for intractable heart failure or aortic thromboembolism. Non-HCM cats were euthanized for a variety of reasons including neoplasia, acute kidney injury, and dementia. HCM was diagnosed either by echocardiography or by necropsy/histopathology or both (Figure 1). Full clinical details including treatment at the time of death were available for all feline cases in this study. Echocardiography was performed either using a GE Vivid-7 ultrasound machine with a 7.5 mHz transducer or where the cat was too unstable, a cage side exam was performed using a GE Vivid-I portable ultrasound machine (GE Systems Ltd., Hatfield, United Kingdom) with a 7.5 mHz transducer. All LV wall thickness measurements were made from 2-D recorded images. Maximal LV wall thickness in diastole was measured on the last frame before mitral valve closure on images where the mitral valve was visible and at the time point in the cardiac cycle of greatest LV internal diameter on images where the mitral valve was not visible. A leading-edge-to-trailing-edge method of measurement was used, being careful to exclude the pericardium, false tendons or papillary muscles, but including the endocardial borders (Lang et al., 2005). A diagnosis of HCM was made if the LV wall thickness exceeded 6 mm in the absence of predisposing factors such as aortic stenosis and systemic arterial hypertension (Fox et al., 1995). A record of the pattern of LV hypertrophy (global or regional) was made (Table 1). Pathological assessment consisted of gross pathology and in the majority of cases also histopathology. Briefly, a semi-quantitative scoring system was used to grade histopathologic criteria as previously described (Wilkie et al., 2015). Morphological lesions assessed included myocyte hypertrophy, myofiber disarray, fibrosis (interstitial, perivascular, replacement, subendocardial), inflammatory cell infiltrate, intramural arteriolosclerosis, myocyte degeneration, and fat infiltration. The certainty of diagnosis (HCM or non-HCM) for each sample used in the study was scored using the following criteria. ^***^HCM or non-HCM heart unequivocally diagnosed by echocardiography and by pathological assessment with appropriate clinical signs. ^**^HCM or non-HCM heart unequivocally diagnosed by either echocardiography or pathological assessment with appropriate clinical signs. ^*^Either equivocal findings on echocardiography and or pathology or neither echocardiography nor pathology performed but the cat must have had appropriate clinical signs. Equivocal findings included, emergency cage side echocardiography which was not detailed enough to allow for accurate assessment of LV morphology and histopathological changes which were considered too minor to be diagnostic.

**Table 1:** Clinical data on feline samples.

**Ref no**.	**Breed**	**Age/Years**	**Gender**	**Immediately frozen at −80**	**Cardiac Physical examination**	**Echocardiography**	**Pathology**	**Reason for Euthanasia**	**Cardiac diagnosis**	**Strength**	**Haploinsufficiency**	**Treatment**
N1	DSH	11.4	F	N (−20 for < 12 h)	No murmur, no arrhythmia, no gallop sound. SBP WNL	Not performed	Not performed	Ureteric Obstruction, Azotemia	Undetermined	^*^		No treatment
N4	Norwegian Forest	10.4	F	Y	No murmur, no arrhythmia, no gallop sound, increased bronchial wheeze. SBP not recorded	Normal cardiac structure	Normal gross and histopathology	Chronic Bronchitis/Low grade pneumonia	Normal	^***^		Inhaled corticosteroid/antibiotics
N7	DLH	13	M	N (−20 < 14 day transferred to RVC on dry ice)	No murmur, no arrhythmia, no gallop sound. SBP WNL	Normal cardiac structure	Normal gross and histopathology	Hepatic Neoplasm	Normal	^***^		No treatment
N8	DLH	5	M	N (−20 < 14 day transferred to RVC on dry ice)	No murmur, no arrhythmia, no gallop sound, SBP WNL	Normal cardiac structure	Normal gross and histopathology	Suspect mediastinal Carcinoma	Normal	^***^		No treatment
N9	DSH	3.5	M	N (−20 < 14 day transferred to RVC on dry ice)	No murmur, no arrhythmia, no gallop sound, SBP WNL	Normal cardiac structure	Normal gross pathology, No myofiber disarray, mild infiltrate of macrophages in LV myocardial interstitium	FeLV related disease	Undetermined but not HCM	^*^		Corticosteroid, Ampicillin
N11	Tonkinese	18.9	F	Y	No murmur, no arrhythmia, no gallop sound, SBP not recorded	Normal cardiac structure	Normal gross and histopathology	pancreatic carcinoma	Normal	^***^		Opioids, Ondansatron
N12	DSH	10.5	M	N (−20 < 14 day transferred to RVC on dry ice)	No murmur, no arrhythmia, no gallop sound, SBP WNL	Normal cardiac structure	Normal Gross and histopathology	Acute kidney injury	Normal	^***^		IV fluid therapy
H1	DSH	1.7	F	Y	Grade IV sternal murmur and arrhythmia. Hypotensive SBP 75 mmHg	Cage side echo—diastolic symmetrical hypertrophy of LV consistent with HCM and enlarged LA	Gross pathology symmetrically thick LV walls—Myofiber disarray 15%, interstitial fibrosis consistent with HCM	Poor prognosis, financial constraints	HCM	^***^		No treatment
H2	Sphynx	9.7	M	Y	Increased respiratory rate, arrhythmia and gallop sound, SBP WNL.	Enlarged left atrium and symmetrically thickened LV walls with one area of infarction of LVFW consistent with ES-HCM	Myofiber disarray 5-10%, multifocal replacement fibrosis including transmural LVFW, interstitial fibrosis, intramural arteriosclerosis—ES-HCM	Refractory congestive heart failure	HCM	^***^		Clopidogrel, furosemide
H3	DSH	8	M	N (—20 for < 12 h)	Increased respiratory rate, hind limb paresis, pulseless and cold to touch, SBP (forelimb) WNL consistent with aortic thromboembolism. Cat diagnosed with HCM 3 years previously by echocardiography at the RVC.	Cage side echo—severe thickening of the LVFW, enlarged left atrium with spontaneous contrast present	Not performed	Suspected aortic thromboembolism	HCM	^***^		Furosemide, LMW heparin
H4	DSH	18.6	F	N (—20 for < 12 h)	Increased respiratory rate, distress and vocalization, hind limb paresis, pulseless and cold to touch consistent with aortic thromboembolism, SBP not recorded	Not performed	Not performed	Suspected aortic thromboembolism/financial constraints	Cardiomyopathy likely but not confirmed	^*^		No treatment
H5	Ragdoll	10	M	N (−20 for < 12 h)	Increased respiratory rate, collapse, arrhythmia, gallop sound, pulmonary crackles. Grade II murmur. Homozygous MYBPC3 R820W. SBP WNL	Enlarged LA with poor systolic function and spontaneous contrast, mild/moderate hypertrophy of the IVS. LV systolic function reduced	Gross pathology—LV hypertrophy particularly affecting IVS, significantly enlarged LA	Chronic congestive heart failure		^***^		
H6	Bengal	4	F	N (−20 for < 12 h)	Increased respiratory rate, hind limb paresis, pulseless and cold to touch consistent with aortic thromboembolism, SBP reduced 100 mmHg	Cage side echo—enlarged LA, focal severely thickened area of basal IVS, cardiomegaly and pulmonary oedema on radiography	Not performed	Suspected aortic thromboembolism	HCM	^**^	—21%	Furosemide, LMW heparin, opioids
H7	DSH	10.8	M	N (−20 for < 12 h)	Increased respiratory rate, Intermittent gallop sound, intermittent arrhythmia, ECG identified VPCs, SBP WNL	Enlarge LA with spontaneous contrast, thickened basilar IVS, and area of thin and hypomotile LVFW consistent with ES-HCM	Not performed	Aortic thromboembolism	HCM	^**^	−30%	Furosemide, aspirin, benazapril
H8	DSH	10.1	M	N (−20 for < 12 h)	Increased respiratory rate and harsh lung sounds, weak peripheral pulses, pulmonary oedema on radiography, signs consistent with aortic thromboembolism, SBP reduced 90 mmHg	Enlarged LA, mild hypertrophy of LV with poor systolic function	Not performed	Aortic thromboembolism and poor cardiac output	HCM	^**^	−22%	Furosemide, clopidogrel, pimobendan
H10	DSH	12.7	M	N (−20 for < 12 h)	Hypothermia, persistent arrhythmia, pelvic limb paresis and cold to touch. SBP not recorded	Cage side echo—enlarged poorly motile LA with thrombus visualized, LV hypomotile with diastolic thickening of IVS	Not performed	Aortic thromboembolism, financial constraints	HCM	^**^	−22%	No treatment
H11	DSH	4.6	M	Y	Increased respiratory rate, crackles over lung fields, arrhythmia. SBP not recorded	Cage side echo—enlarge LA and symmetrical LV hypertrophy	Gross pathology symmetrical LV hypertrophy. Myofiber disarray 30%, moderate interstitial fibrosis, intramural arteriosclerosis, replacement fibrosis papillary muscles	Poor prognosis, financial constraints	HCM	^***^		Furosemide
H12	BSH	3	M	Y	Increased respiratory rate—pleural effusion on thoracic ultrasound. SBP low 110 mmHg	Cage side echo—Severe left ventricular hypertrophy, very dilated left atrium, very poor left atrial function	Gross pathology symmetrical severe LV hypertrophy. Myofiber disarray, moderate interstitial fibrosis and intramural arteriosclerosis	Refractory congestive HF and azotemia	HCM	^***^		Furosemide, oxygen
H13	DSH	14	F	N (−20 < 14 day transferred to RVC on dry ice)	Distress, increased respiratory rate, open mouth breathing, arrhythmia. SBP not recorded	Not performed, brief thoracic U/S revealed pleural effusion and enlarged atria—ventricular morphology not fully assessed.	Gross pathology moderate LV hypertrophy. Endocardial fibrosis and myocardial disarray on histopathology consistent with HCM	Refractory congestive HF	HCM	^***^		Thoracentesis furosemide oxygen
H14	DSH	4	M	Y	Distressed, increased respiratory rate and effort. Tachycardia and gallop sound, bilateral pulmonary crackles. Both hind limbs had no motor function and were cold to the touch no femoral pulse was palpable. SBP was not recorded	Enlarged LA with reduced systolic function, severe hypertrophy of LVFW adequate LV systolic function, incidental false tendon, small volume pericardial effusion.	Not performed	Congestive HF and suspected aortic thromboembolism, financial constraints	HCM	^**^	−44%	Furosemide
H15	DSH	12	M	Y	Distressed, hypothermia, increased respiratory rate increased and effort, open mouth breathing. Arrhythmia detected. Both hind limbs had no motor function and were cold to the touch no femoral pulse was palpable. SPB was not recorded	Not performed	Not performed	Suspected aortic thromboembolism and severe CHF, poor prognosis	HCM/other cardiomyopathy	^*^		None given
H16	Maine Coone	11	M	N (−20 < 14 day transferred to RVC on dry ice)	Increased respiratory rate, pulmonary crackles. SBP WNL	Symmetrical moderate LV hypertrophy, LA dilation and poor LA function	Myofiber disarray 5%, interstitial fibrosis, intramural arteriosclerosis consistent with HCM (incidental solid pulmonary carcinoma) identified at PM	CHF, financial constraints	HCM	^***^	−19%	
H17	Ragdoll	7	F	N (−20 < 14 day transferred to RVC on dry ice)	Collapse/seizure—cardiac auscultation unremarkable. Hypotensive SBP 80 mmHg	Not performed	Gross pathology moderate symmetrical LV hypertrophy. Histopathology Myofiber disarray <5%, mild interstitial fibrosis, occasional intramural arteriosclerosis equivocal for HCM	Chronic lethargy/collapse	HCM	^**^		
H18	DSH	8	F	N (−20 < 14 day transferred to RVC on dry ice)	Cardiac auscultation unremarkable. SBP WNL	Not performed	Gross pathology mild LV hypertrophy. Histopathology multifocal myocyte hypertrophy and mild myofiber disarray. Histopathology equivocal for HCM	Pneumothorax/financial constraints	HCM	^*^		
H19	DSH	7	F	N (−20 < 14 day transferred to RVC on dry ice)	Increased respiratory rate, tachycardia. SBP not recorded	Cage side—pleural effusion and enlarged atria on U/S. LV morphology not determined	Not performed	Chronic Chylothorax and uncontrolled hyperthyroidism	HCM/hyperthyroid cardiomyopathy	^*^		

N1-N12 describe myocardial samples from cat without structural heart disease and H1-H19 describe myocardial samples from cats with HCM. Cat breed is shown in column 2, pedigree cat breeds are shown in green. BSH = British Short Hair, DSH/DLH = Domestic Short/Long Hair Based on clinical examination, echocardiography, histology and cause of death, or euthanasia we scored the diagnosis. Overall certainty of diagnosis is given as = probable, ^**^ = highly likely, ^***^ = definitive; see methods section for clinical criteria. The yellow shaded cats were studied by in vitro motility assay (IVMA). The MYBPC3 R820W mutant Ragdoll cat is sample H5.

**Diagnostic criteria:**

*** *HCM or normal heart diagnosed by echocardiography (end diastolic LV wall thickness > 6 mm—see Section Materials and Methods for further details) and by pathological assessment with appropriate clinical signs*.

** *HCM or normal heart diagnosed by either echocardiography or pathological assessment with appropriate clinical signs*.

* *Either equivocal findings on echocardiography and or pathology or neither echocardiography nor pathology performed the cat must have had appropriate clinical signs*.

### Myectomy and donor collection methods

Human myocardial samples were obtained from patients with hypertrophic obstructive cardiomyopathy (HOCM) undergoing surgical septal myectomy for relief of left ventricular outflow tract obstruction (LVOTO). Local ethical approval was obtained from University College London Hospitals and the Brompton, Harefield, and NHLI ethics committees for collection and use of tissue samples (for clinical details, see Supplementary Table S1).

Donor hearts had no history of cardiac disease and normal ECG and ventricular function and were obtained when no suitable transplant recipient was found. Approval was granted by the Human Research Ethics Committees of both the University of Sydney (Protocol No. 2814) and St. Vincent's Hospital (Protocol No. H91/048/1a) for the collection and distribution of the human heart samples and by the NHS National Research Ethics Service, South West London REC3 (10/H0803/147) for study of the samples. Patients gave written consent with PIS approved by the relevant ethical committee. All samples are anonymised. The investigations conform to the principles of the Declaration of Helsinki. These samples have been previously described (Messer et al., 2009, 2016; Copeland et al., 2010; Bayliss et al., 2012).

### Identification of *MYBPC3* A31P and R820W mutations

The known cat HCM-causing mutations were identified using a PCR based assay to test for the mutation using DNA extracted from either blood pellets or buccal mucosal swabs. The tests were performed by Langford Diagnostic Laboratories, University of Bristol Faculty of Veterinary Medicine.

### Isolation of myofibrils, MyBP-C quantification

Whole tissue extracts were obtained for gel electrophoresis using T-Per Protein extraction reagent (Pierce, Thermo Scientific) including 1 μg/ml E64, chymostatin, and leupeptin protease inhibitors according to the manufacturer's protocol. The myofibrillar fraction of heart muscle was prepared by our standard protocol (Messer et al., 2007) and analyzed by SDS-PAGE (Bio-Rad Criterion Criterion TGX™ gradient gel 4–15%) stained with SYPRO Ruby protein stain or in Western blots using mouse anti-MyBP-C F5 (GE Healthcare) (1/1,000 dilution), anti-TnI clone 14G5 (Abcam), and mouse EA-53 anti-α-actinin antibody (Sigma) (1/2,000), visualized with ECL Western Blotting Detection Reagent (GE Healthcare).

The level of phosphorylation of troponin I (TnI) and MyBP-C in myofibrils and troponin was measured by phosphate affinity SDS-PAGE as shown in Supplementary Data 1 using the methods of Messer et al. (2009) for TnI and Copeland et al. (2010) for MyBP-C.

Troponin was isolated from cat heart muscle myofibrils, using an anti-cTnI monoclonal antibody affinity column as described by Messer et al. (2007). This yields pure and active troponin, which is usable for 3 days in an *in vitro* motility assay (IVMA). Where appropriate, troponin was dephosphorylated by treatment with shrimp alkaline phosphatase (Sigma, P9088) and phosphorylation levels were increased by treatment with protein kinase A (PKA) catalytic subunit (Sigma, P2645-400). Recombinant human cardiac troponin T was exchanged into cat heart troponin following the procedures and controls previously described (Messer et al., 2007, 2016; Bayliss et al., 2012; Memo et al., 2013).

### Quantitative *in vitro* motility assay (IVMA)

Thin filaments were reconstituted with 10 nM rabbit skeletal or mouse cardiac muscle α-actin (labeled with TRITC phalloidin), tropomyosin (60 nM), and troponin (60 nM) to study Ca^2+^-regulation of filament motility by the quantitative *in vitro* motility assay (IVMA; Fraser and Marston, 1995; Messer et al., 2007). Thin filament movement over a bed of immobilized rabbit fast skeletal muscle heavy meromyosin (100 μg/ml) was compared in paired motility chambers in which troponin varied by a single factor (mutation, phosphorylation state, or treatment with drug). The temperature was set to 29°C. Filament movement was recorded and analyzed as previously described (Marston et al., 1996) yielding two parameters; the fraction of filaments moving and the speed of moving filaments. These parameters were measured over a range of Ca^2+^ concentrations to generate Ca^2+^-activation curves as shown previously (Memo et al., 2013; Papadaki et al., 2015). The data were fitted to the four-variable Hill equation to yield a value for EC_50_ and n_*H*_. EC_50_-values from replicate experiments were analyzed by paired *t*-test since the distribution of EC_50_ has been shown to be normal.

## Results

### Cat heart samples

We sourced 18 cardiac samples from cats treated at the RVC and 7 from collaborating veterinary practices. Non-HCM samples were collected from patients euthanized for non-cardiac reasons. Samples were cooled (QMHA: −80°C, collaborating practices: −20°C) and stored at −80°C or in liquid nitrogen at below −160°C. All available data for the samples is collated in Table 1. We made a careful examination of the clinical records, particularly echocardiography and histology data, and scored the strength of HCM or non-HCM diagnosis for this cat population using the criteria described in the methods section. Examples of echocardiography and histological sections are shown in Figure 1 and additional echocardiography scans are shown in Supplementary Data 3 and in the Supplementary movies. All the samples were studied by gel electrophoresis and eight HCM cats and five non-HCM cats with the highest certainty of diagnosis were selected for further investigation by *in vitro* motility assay (IVMA).

The majority of the cats studied were outbred domestic shorthaired animals but we also studied samples from pedigree cats with HCM including Ragdoll, Maine Coon, British Short Hair, and Norwegian Forest breeds with established high prevalence of inherited HCM. The homozygous *MYBPC3* R820W mutation was identified in one Ragdoll cat sample.

### Cat heart contractile proteins

**Figure 2:**
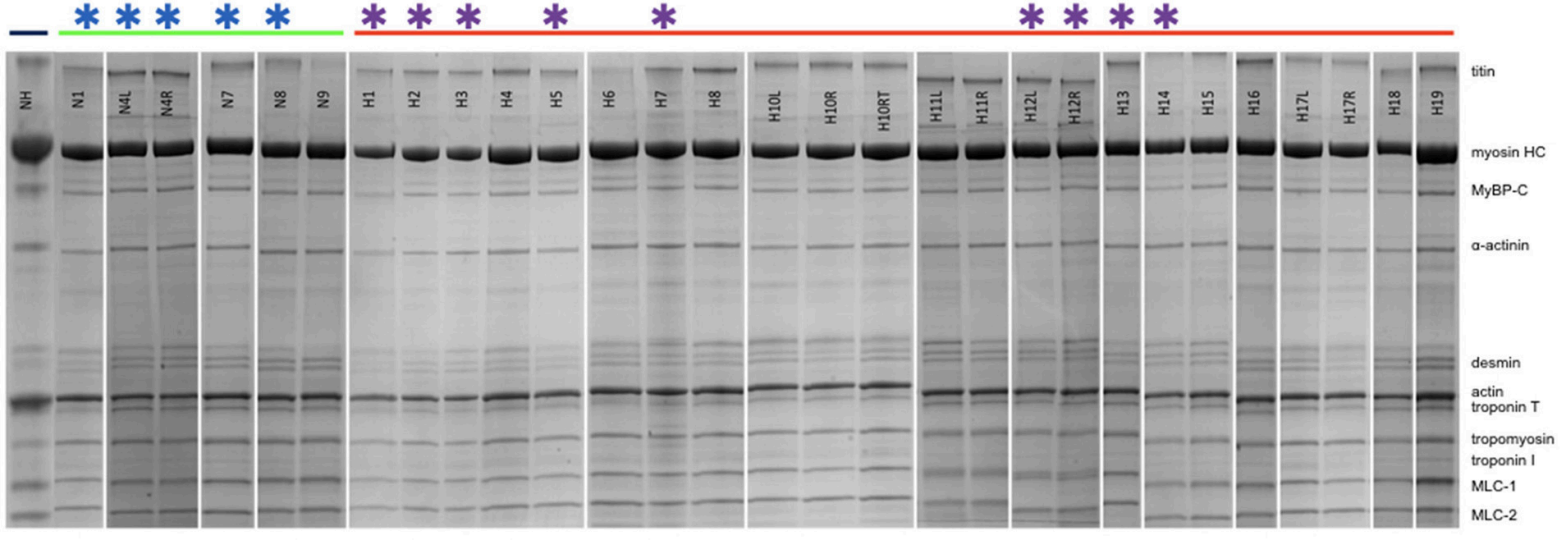
SDS-PAGE of cat and human myofibrillar fraction. NH is a human heart sample for comparison. The green bar shows the non-HCM cat myofibrils (N1-N9) and the red bar shows the HCM cat myofibrils (H1-H19) (see Table 1). All myofibrils are from left ventricular heart samples, except for H10, H11, H12, and H17 where both left (L) and right (R) ventricular samples are shown. H10RT is a portion of the H10L sample, which was left for 90 min post-excision before freezing. Stars show samples used for IVMA studies.

Figure 2 shows examples of SDS-PAGE of the myofibrils extracted from our collection of cat hearts in comparison with a human heart sample (NH). All the expected contractile proteins were identified in the cat samples in the appropriate proportions. Observation of the feline band patterns showed no signs of protein degradation (band streaking, high background or fragmented bands). H10L and H10RT are two left ventricular samples from the same cat with H10RT left at room temperature for 90 min post-excision before freezing. Comparison of the band patterns showed there was no indication of protein degradation after this treatment.

Since the only HCM-causing mutations so far identified in cats are in the *MYBPC3* gene and because most *MYBPC3* mutations associated with HCM in humans result in haploinsufficiency, we determined the quantity of MyBP-C relative to the constitutive protein α-actinin.

**Figure 3:**
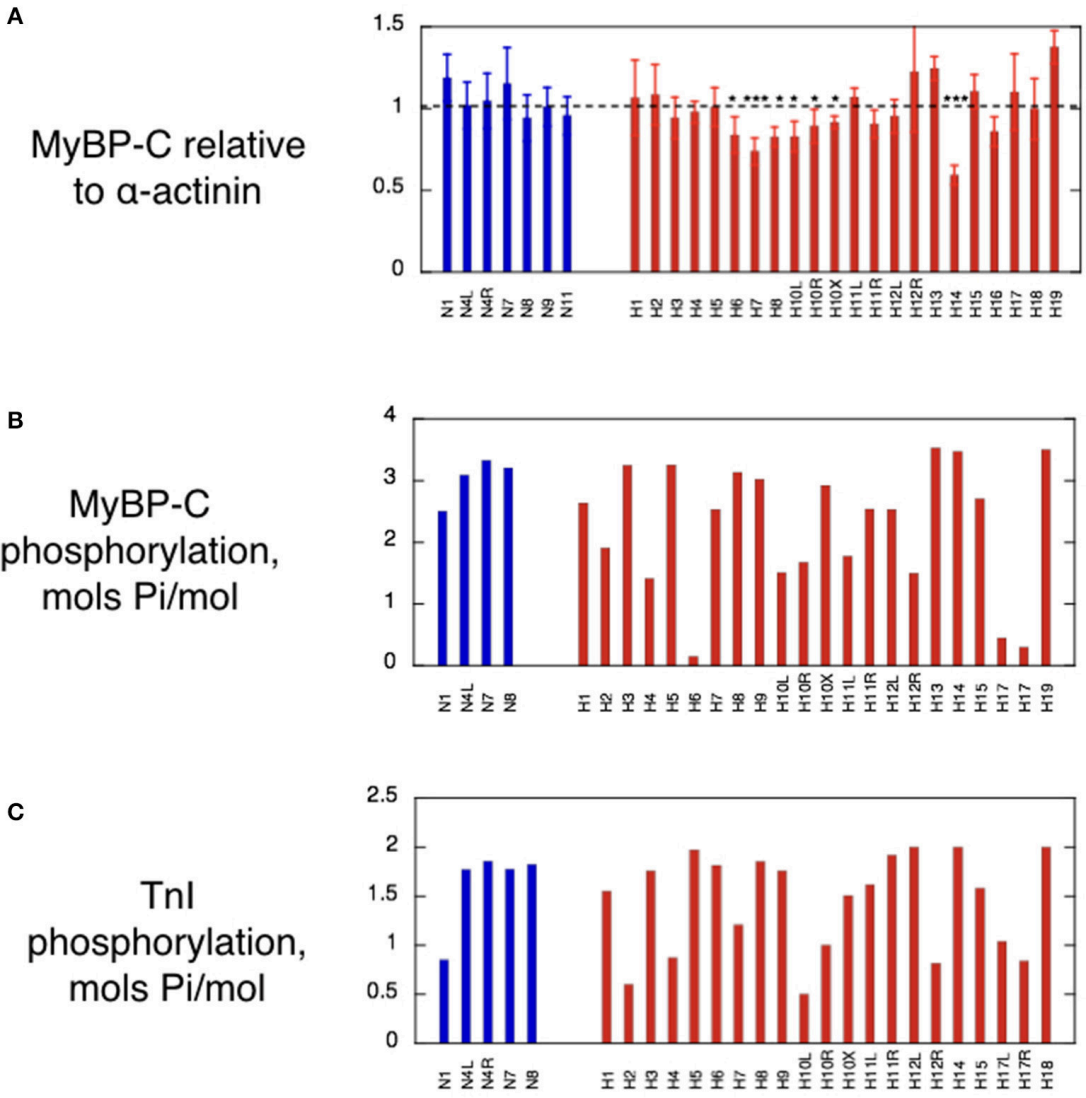
MyBP-C content, MyBP-C phosphorylation and Troponin I phosphorylation in cat heart muscle samples. **(A)** Histogram showing the MyBP-C content of the cat samples based on analysis of multiple SDS-PAGE gels similar to those in Figure 2. The normalized ratio of MyBP-C band to α-actinin is plotted as the mean and standard deviation of at least 6 replicate lanes for each sample. Non-HCM and HCM cat samples were compared by one-way ANOVA and 5 samples showed significant haploinsuffficiency. ^*^p ≤ 0.05, ^**^p ≤ 0.01, ^***^p ≤ 0.001. **(B)** Histogram of the phosphorylation levels of MyBP-C measured by phosphate affinity SDS-PAGE using the technique of Copeland et al. (2010). See supplement for details of method. **(C)** Histogram of troponin I phosphorylation levels in cat myofibrils measured by phosphate affinity SDS-PAGE using the technique of Messer et al. (2009). See supplement for details of method. Blue bars, non-HCM cat samples; red bars, HCM cat samples.

The average cMyBP-C/α-actinin ratio in each lane was normalized against the average value for the non-HCM cats. In the HCM cat samples we found that five samples (H6, H7, H8, H10, and H14) have significantly lower cMyBP-C content when compared to non-HCM samples (Figure 3A). In addition, H16 exhibits a marked reduction approaching significance in one-way ANOVA. The percentage decrease of cMyBP-C was around 20% in the majority of deficient samples (H6, H8, H10, and H16), but two samples had a much lower content, H7 (30%) and H14 (44%) (Table 2). Of the deficient samples 4 were domestic shorthair (DSH), one (H6) was a Bengal, and one (H16) was a Maine Coon (without the *MYBPC3* A31P mutation). Interestingly, H5 (the Ragdoll cat genotyped to be homozygous for the *MYBPC3* R820W mutation) did not exhibit a cMyBP-C deficiency, even when compared to H17 (a Ragdoll cat without the *MYBPC3* R820W mutation) in a one-way *T*-test. In Western blots, none of the samples showed any lower molecular weight bands that could be truncated peptides (Supplementary Data 2).

**Table 2:** Analysis of MyBP-C haploinsufficiency in HCM cat heart muscle.

**Sample**	**Mean cMyBP-C relative to non-HCM**	**Percentage deficiency of MyBP-C**	**Significance (*p*)**	**Repeats (*n*)**
H6	0.79	21	<0.05	12
H7	0.70	30	<0.001	6
H8	0.78	22	<0.05	6
H10	0.78	22	<0.05	6
H14	0.56	44	<0.05	6
H16	0.81	19	<0.001	6

Non-HCM and HCM cat samples were compared by one-way ANOVA and 5 samples showed significant haploinsuffficiency. ^*^ p ≤ 0.05, ^**^p ≤ 0.01, ^***^p ≤ 0.001. H16 is close to significance in the ANOVA test and is significant in a paired t-test when compared with non-HCM samples in the same gel.

### Phosphorylation level of MyBP-C and TnI is not significantly changed in the myocardium of HCM cats

Protein phosphorylation levels were determined by phosphate affinity SDS-PAGE. Cat cMyBP-C phosphorylation data is presented in Figure 3B. The overall phosphorylation of the non-HCM cat samples was 3.03 ± 0.37 mols Pi/mol (*n* = 4), which compares with 2.70 ± 0.07 mols Pi/mol reported in human donor heart muscle. In human myectomy samples, MyBP-C phosphorylation is substantially reduced (1.05 ± 0.13 mols Pi/mol; Messer et al., 2009).

Although some cat HCM samples, notably H6 and H17, appeared dephosphorylated, the majority of cat HCM samples had phosphorylation levels comparable with non-HCM cat samples. When all the cats were averaged, there was no significant overall reduction of cMyBP-C phosphorylation in the HCM samples (mean = 2.32 ± 0.95 mols Pi/mol, *n* = 17, *p* = 0.22) in contrast to the human myectomy samples.

Phosphorylation assessments of feline troponin I (TnI) showed similar results to MyBP-C. Certain samples appeared dephosphorylated (H2 and H10), but there was no overall significant difference between the normal and hypertrophic groups (Non-HCM: mean = 1.49 ± 0.17 mols Pi/mol TnI, *n* = 8; HCM: mean = 1.46 ± 0.12 mols Pi/mol TnI, *n* = 18, *p* = 0.874; Figure 3C). This is very different from human heart where donor heart muscle has a phosphorylation level similar to non-HCM cat (1.62 ± 0.06 mols Pi/mol TnI) but myectomy samples have a very low level of phosphorylation (0.26 ± 0.02 mols Pi/mol TnI) (Messer et al., 2009; Bayliss et al., 2012). The low levels of TnI or MyBP-C phosphorylation in some of the cat samples may be a consequence of the post-mortem treatment of the cat hearts although we did not detect any correlation between the time of storage at −20°C prior to −80°C freezing and the level of TnI or MyBP-C phosphorylation.

### Thin filaments from HCM cats have higher Ca^2+^-sensitivity than non-HCM cats

To study Ca^2+^ regulation of contraction, we extracted troponin from cat hearts using the protocols we have developed for human heart troponin. Yields were comparable to human and the troponin was fully competent at regulating thin filaments in the quantitative *in vitro* motility assay (IVMA). We investigated the Ragdoll (*MYBPC3* R820W mutation) (H5) cat heart troponin in detail and also studied troponin from 7 other cats that had a high confidence of HCM diagnosis (see Table 1).

**Figure 4:**
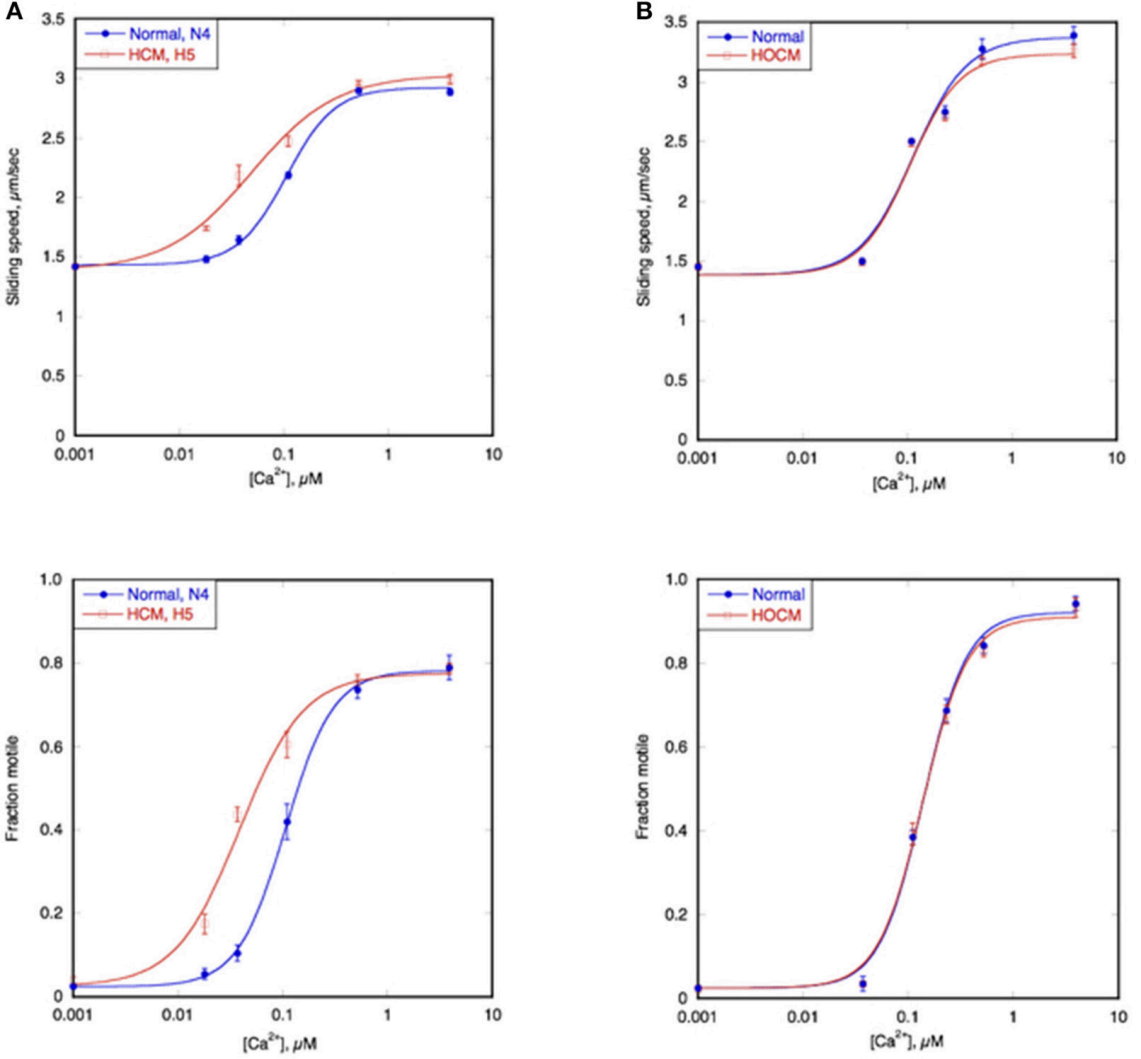
Ca^2+^-sensitivity of HCM cat and human compared with non-HCM control. The sliding speed (top graphs) and fraction of filaments motile (bottom graphs), measured in paired experiments by IVMA is plotted against [Ca^2+^] for representative single experiments. HCM and non-HCM samples are compared. **(A)** Example of cat heart troponin; cTnI in both N4 and H5 troponins are fully phosphorylated. **(B)** example of human heart troponin; cTnI in normal troponin is fully phosphorylated whilst HOCM troponin is unphosphorylated. Blue lines and points, non-HCM troponin (cat N4 and human NK), red lines and points, HCM troponin (cat H5 and human MR). The Hill equation is fitted to the data (solid lines) to yield values of EC_50_ and n_H_. The mean values of replicate experiments are shown in Figure 5 for cats and Supplementary Table S2 for humans.

Figure 4A shows an example of the Ca^2+^ titration curves of HCM cat H5 compared to a non-HCM cat, N4; both had a naturally high level of troponin phosphorylation (Figure 3C). The left shifted Ca^2+^ curves of both sliding speed and fraction motile and the calculated lower EC_50_ indicates that the HCM cat has a 1.5–2.0 fold higher Ca^2+^-sensitivity than non-HCM cat troponin. This was confirmed with a total of 6 H5 troponin preparations, compared with 2 different non-HCM troponins (mean non-HCM EC_50_ = 0.094 ± 0.005 μM, mean H5 EC_50_ = 0.045 ± 0.004, mean EC_50_ non-HCM/EC_50_ H5 = 2.19 ± 0.27, *p* = 0.008).

**Figure 5:**
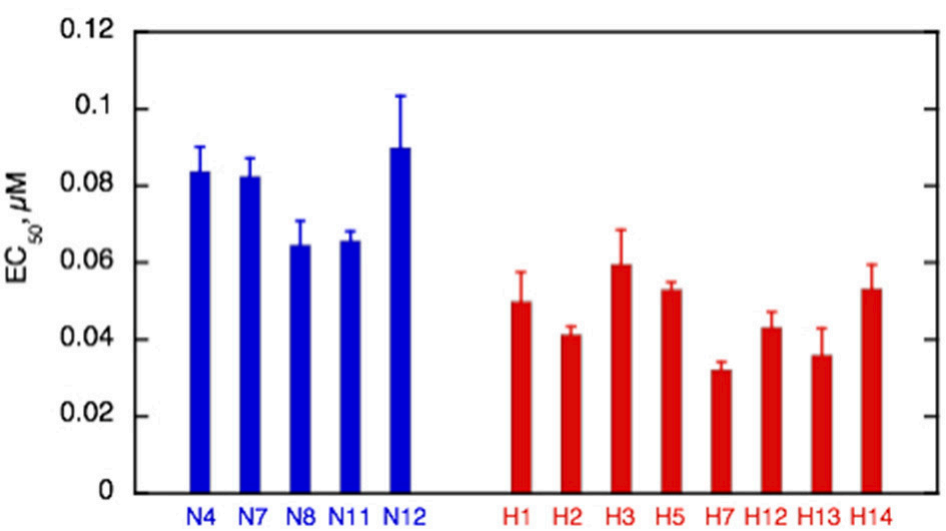
Summary of EC_50_ in non-HCM and HCM cat troponin. Mean EC_50_ ± sem is shown for at least three replicate determinations of Ca^2+^-sensitivity for non-HCM cat troponin (blue) and HCM cat troponin (red) as shown in Figure 3A. All the HCM cat samples have a higher Ca^2+^-sensitivity than non-HCM samples. See Supplementary Table S6 for full details.

Ca^2+^-sensitivity of HCM cat troponin was tested in seven further cats, including two that showed MyBP-C haploinsufficiency (H7 and H14), all of which showed a higher Ca^2+^-sensitivity than non-HCM troponin in paired (non-HCM + HCM) assays combining fraction motile and sliding speed at similarly high levels of troponin phosphorylation (mean non-HCM EC_50_ = 0.090 ± 0.005 μM, mean all HCM troponin EC_50_ = 0.046 ± 0.003, mean EC_50_ non-HCM/EC_50_ HCM = 2.05 ± 0.13, *n* = 7, *p* < 0.0001), see Figure 5 and Table 3. There were no significant differences in n_H_ and maximum sliding speed between non-HCM and HCM cat troponin.

**Table 3:** Effects of mutations and phosphorylation on Ca^2+^-sensitivity of thin filaments containing troponin from HCM cat hearts.

**CAT**		**All Non-HCM**	**H1**	**H2**	**H3**	**H5**	**H7**	**H12**	**H13**	**H14**	**All HCM**
EC_50_, μM ±*SEM, n* and *p*-value (Unpaired *t*-test relative to non-HCM)	Sliding speed	0.092 ± 0.006	0.060 ± 0.010	0.043 ± 0.004	0.083 ± 0.012	0.056 ± 0.003	0.037 ± 0.002	0.054 ± 0.002	0.048 ± 0.015	0.054 ± 0.010	0.057 ± 0.003
		*n =* 24	*n =* 2	*n =* 3	*n =* 6	*n =* 19	*n =* 3	*n =* 4	*n =* 2	*n =* 5	*n =* 44
			*p* = 0.136	*p* = 0.0080	*p* = 0.49	*p* < 0.0001	*p* = 0.0032	*p* = 0.015	*p* = 0.047	*p* = 0.0095	*p* < 0.0001
	Fraction motile	0.072 ± 0.004	0.040 ± 0.007	0.039 ± 0.004	0.036 ± 0.001	0.050 ± 0.002	0.029 ± 0.001	0.033 ± 0.003	0.028 ± 0.002	0.053 ± 0.008	0.043 ± 0.002
		*n =* 26	*n =* 2	*n =* 3	*n =* 6	*n =* 19	*n =* 4	*n =* 4	*n =* 3	*n =* 5	*n =* 46
				*p* = 0.011	*p* = 0.0002	*p* < 0.0001	*p* = 0.0003	*p* = 0.0008	*p* = 0.0011	*p* = 0.061	*p* < 0.0001
EC_50_P/unP ± SEM, *n* and *p*-value (Single values compared with 1)	Sliding speed	1.833 ± 0.112	1.091 ± 0.118	0.988 ± 0.049	1.068 ± 0.088	1.098 ± 0.033	0.957 ± 0.021	1.010 ± 0.112	0.991	0.997 ± 0.122	1.050 ± 0.023
		*n =* 8	*n =* 2	*n =* 3	*n =* 3	*n =* 10	*n =* 2	*n =* 2	*n =* 1	*n =* 2	*n =* 25
		*p* = 0.0001		*p* = 0.82	*p* = 0.52	*p* = 0.015					*p* = 0.037
	Fraction motile	1.796 ± 0.055	1.006 ± 0.080	0.960 ± 0.054	1.001 ± 0.015	1.113 ± 0.048	1.051 ± 0.048	0.935 ± 0.022	1.048 ± 0.013	0.838 ± 0.0023	1.030 ± 0.026
		*n =* 9	*n =* 2	*n =* 3	*n =* 3	*n =* 10	*n =* 2	*n =* 2	*n =* 2	*n =* 2	*n =* 26
		*p* < 0.0001		*p* = 0.53	*p* = 0.94	*p* = 0.044					*p* = 0.26
**100** μ**M EGCG** EC_50_ P/unP ± SEM, *n* and *p*-value (Unpaired *t*-test relative to untreated)	Sliding speed	2.32				2.197 ± 0.158			3.377	1.403	2.261 ± 0.277
		*n =* 1				*n =* 4			*n =* 1	*n =* 1	*n =* 6
		–				*p* < 0.0001			–	–	*p* < 0.0001
	Fraction motile	1.54				1.898 ± 0.225			1.927	1.313	1.805 ± 0.173
		*n =* 1				*n =* 4			*n =* 1	*n =* 1	*n =* 6
		–				*p* = 0.00027			–	–	*p* < 0.0001
**TnT exchange** EC_50_ P/unP ± SEM, *n* and *p*-value (Unpaired *t*-test relative to untreated)	Sliding speed					1.696 ± 0.036					
						*n =* 3					
						*p* < 0.0001					
	Fraction motile					1.644 ± 0.052					
						*n =* 3					
						*p* = 0.00016					

*The EC_50_ of thin filaments from non-HCM and HCM cats is compared and the effect of TnI phosphorylation on EC_50_ is plotted for a series of different HCM cat samples, using IVMA. The last two rows show how coupling can be restored by exchange of TnT with wild-type (human sequence) TnT or by EGCG. P-values are given using Students's t-test for HCM compared with non-HCM or for the ratio of EC_50_ in phosphorylated/unphosphorylated troponin compared with 1. Full details of the IVMA experiments are summarized in Supplementary Table S6*.

### Modulation of cat troponin Ca^2+^-sensitivity by TnI phosphorylation

**Figure 6:**
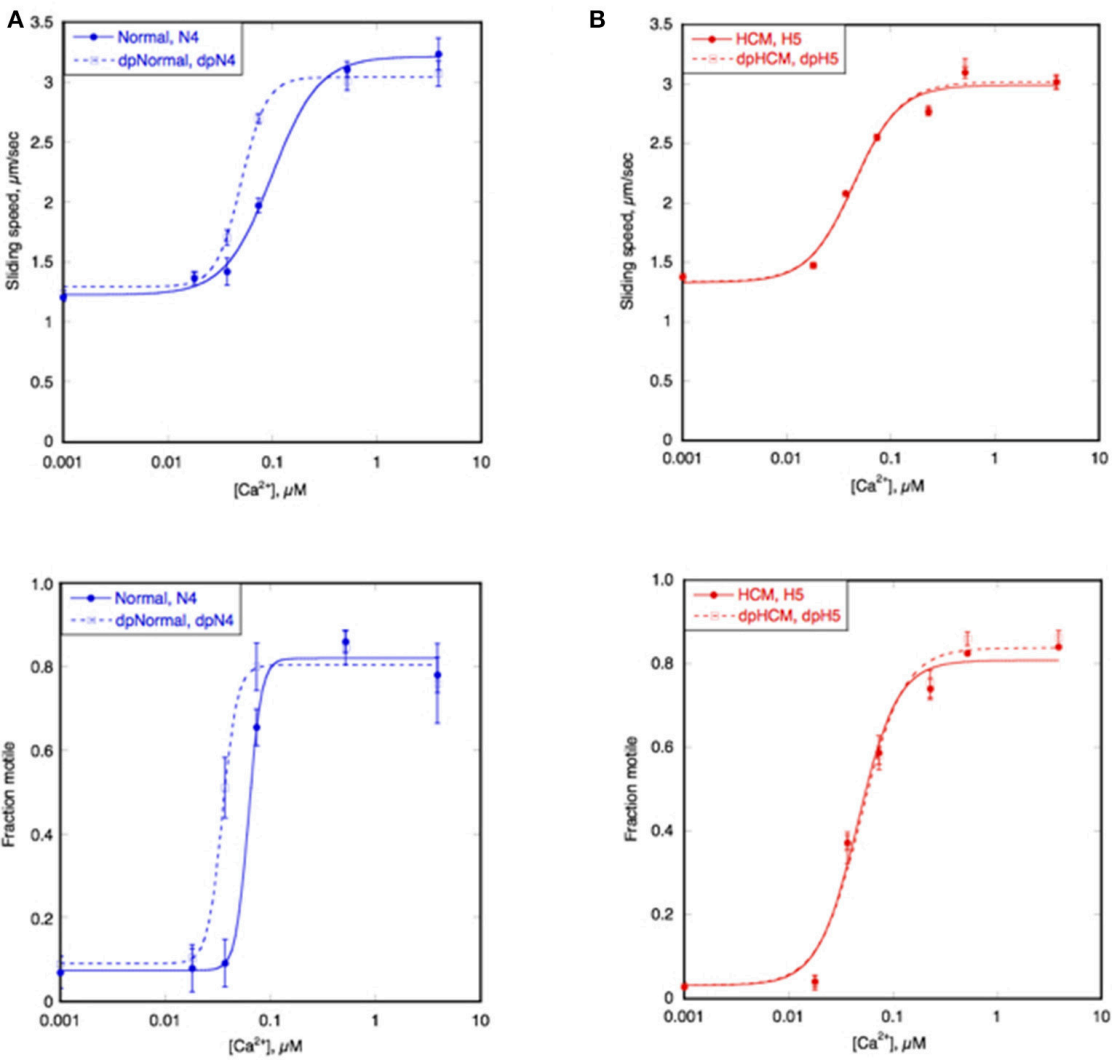
Modulation of Ca^2+^-sensitivity by TnI phosphorylation in non-HCM and HCM cat. Sliding speed (top graphs) and fraction of filaments motile (bottom graphs) was measured with phosphorylated and unphosphorylated troponin by IVMA. Ca^2+^ dependence of motility is plotted against [Ca^2+^] for representative single experiments. **(A)** Phosphorylated and unphosphorylated non-HCM cat troponin (N4), **(B)** phosphorylated and unphosphorylated HCM cat troponin (H5). The Hill equation is fitted to the data to yield values of EC_50_ and n_H_ Solid lines, phosphorylated; dotted lines, unphosphorylated. The mean ratios of EC_50_ values from replicate experiments is shown in Figure 6 and Table 3. Full data is shown in Supplementary Table S3.

**Figure 7:**
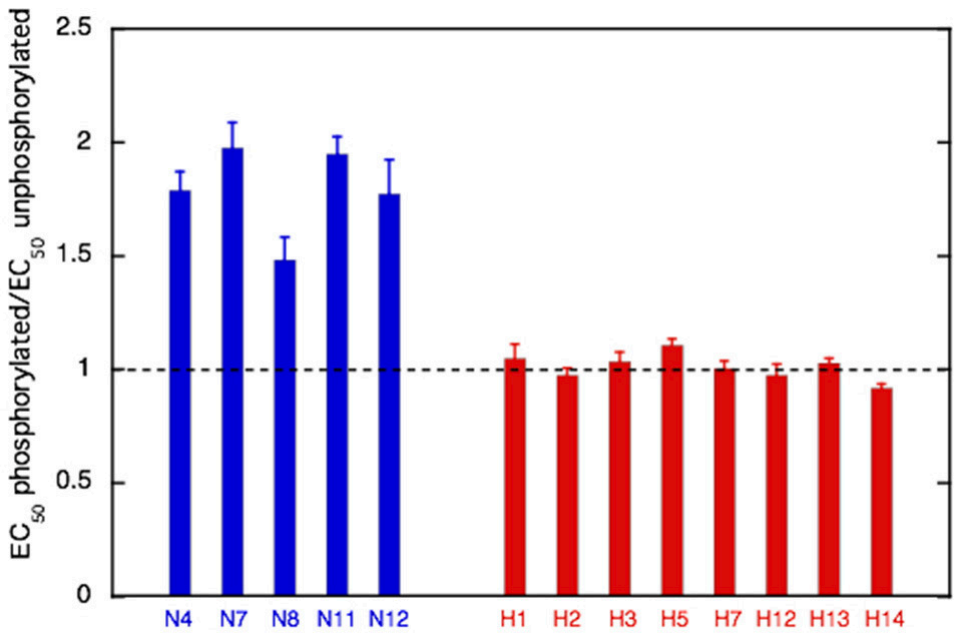
Effect of phosphorylation on Ca^2+^-sensitivity in non-HCM and HCM cat. The mean ratio of EC_50_ phosphorylated:EC_50_ unphosphorylated ± sem is plotted for at least three replicate experiments for non-HCM cat troponin (blue) and HCM cat troponin (red). A ratio of 1 (dotted line) indicates uncoupling. Full data is shown in Supplementary Table S3.

In man and mouse, the Ca^2+^-sensitivity of normal heart muscle troponin is modulated by TnI phosphorylation at Ser 22 and 23 by PKA. This was also true for the non-HCM cat heart samples. Phosphorylation levels were manipulated by dephosphorylating highly phosphorylated troponin and treating unphosphorylated troponin with PKA+ATP. Figure 6A shows the Ca^2+^-activation curve for a typical non-HCM cat troponin in the phosphorylated and unphosphorylated states; there is an ~2-fold increase in EC_50_ upon phosphorylation. The same pattern of results was found with all five cat hearts tested (see Figure 7 and Table 2).

We have frequently found that the Ca^2+^-sensitivity of troponin extracted from the heart muscle of patients with HCM obtained from myectomy operations is not modulated by phosphorylation (Supplementary Table S3; Bayliss et al., 2012; Messer et al., 2016). This also occurred in our HCM cat troponin samples; Figure 6B illustrates this for cat H5. The insensitivity of Ca^2+^-sensitivity to the level of TnI phosphorylation was found for every HCM cat troponin, irrespective of the causative mutation (Figure 7 and Table 3).

### Blunted modulation of Ca^2+^-sensitivity by TnI phosphorylation in HCM cats can be restored by adding EGCG

In studies using human and mouse heart muscle we found that coupling could be restored to HCM thin filaments by adding small molecules such as Epigallocatechin-3-gallate (EGCG) and Silybin (Bayliss et al., 2012; Papadaki et al., 2015; Messer et al., 2016). We tested whether recoupling could be observed in cat heart troponin.

**Figure 8:**
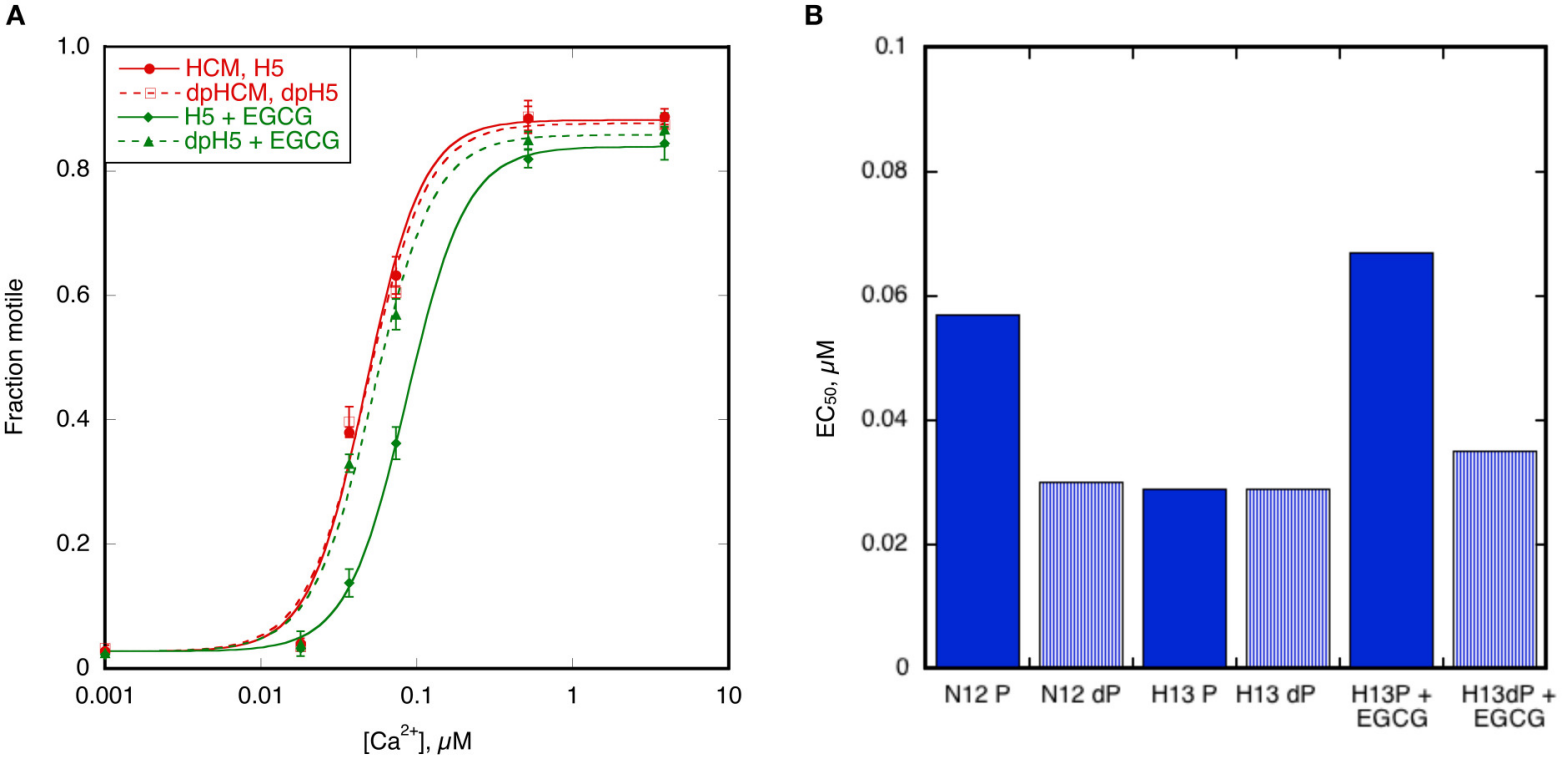
Recoupling of HCM cat thin filaments by EGCG. **(A)** The effect of 100 μM EGCG on cat HCM sample H5 measured by IVMA. The Hill equation is fitted to the data to yield values of EC_50_ and n_*H*._ Solid symbols and lines, phosphorylated; open symbols and dotted lines, unphosphorylated. Red points and lines are in the absence and green points and lines are in the presence of EGCG. EGCG reduces the Ca^2+^-sensitivity of phosphorylated but not unphosphorylated H5 troponin and thus restores coupling. **(B)** Demonstration of complete restoration of coupling by EGCG. The figure shows the phosphorylated and unphosphorylated EC_50_ of the non-HCM cat sample N12 (coupled) and the HCM cat sample H13 (uncoupled). Addition of EGCG to H13 restores phosphorylation-dependence of EC_50_ to H13. Full data in Supplementary Table S5.

When 100 μM EGCG was added to troponin from H5 cat heart it restored coupling in reconstituted thin filaments measured by IVMA (Figure 8A). EGCG did not affect the EC_50_ of unphosphorylated H5 troponin but it increased the EC_50_ of phosphorylated H5 troponin, thus restoring EC_50_ to the values present in the non-HCM cat. The change in EC_50_ on phosphorylation in the presence of EGCG was 2.21 ± 0.16 fold in four separate assays with H5. EGCG also recoupled troponin from H13 and H14 cat hearts by a similar amount (see Table 3). Figure 8B shows the restoration of EC_50_ to normal by EGCG with H13.

In previous studies of HCM in human myectomy samples we observed that it was possible to restore coupling by replacing troponin T (TnT) with wild-type (human) recombinant TnT. We tested whether this also occurred with HCM cat troponin. When we replaced TnT in troponin from the Ragdoll *MYBPC3* R820W H5 cat heart with wild-type (human) recombinant TnT by an exchange reaction we found that the modulation of Ca^2+^-sensitivity by TnI phosphorylation was restored (Supplement S3 and Table 3). The change in EC_50_ on phosphorylation was 1.61 ± 0.01 fold, comparable with the same experiment in HCM human heart troponin (1.85 ± 0.13 fold) and with the phosphorylation effect on EC_50_ in non-HCM cat (Figure 6).

## Discussion

HCM in cats has been recognized for over 30 years and its parallels with HCM in humans have been previously described (Kittleson et al., 1999; Maron and Fox, 2015). HCM in humans is a highly complex and heterogeneous disease at the genetic, cellular, and clinical level. Disease-causing mutations in myosin heavy chain 7 (*MYH7*) and cardiac myosin binding protein C (*MYBPC3*) genes, account for ~70% of identified pathogenic alleles (Maron et al., 2006). Pathogenic alleles that do not encode for sarcomeric proteins have also been identified in some HCM patients however their role remains controversial (Cecconi et al., 2016; Walsh et al., 2017). Mutations in genes responsible for metabolic storage disorders with a clinical presentation and pattern of left-ventricular hypertrophy similar to HCM are also recognized. Furthermore, pathogenic alleles have not been identified in 28–40% of HCM patients with a family history of HCM and 50–90% of sporadic HCM cases (Cecconi et al., 2016). Despite this complexity, mutations in sarcomeric proteins remain the most established and well-studied cause of HCM in humans.

The role of genetic mutations in feline HCM is less well-characterized since to date only two causative mutation have been identified, both in *MYBPC3* (Meurs et al., 2007; Longeri et al., 2013) and the prevalence of phenocopies and moderator genes remains unclear. In the absence of substantial genetic information, we postulated that exploration of feline HCM at the sarcomeric level may identify common regulatory characteristics driving the disease phenotype between species.

In this study, we have investigated left ventricular tissue from 18 cats diagnosed with HCM, including one Ragdoll cat heart with a homozygous *MYBPC3* R820W mutation, in comparison with seven non-HCM cat hearts. Tissue was obtained immediately following euthanasia with owner consent. The cat hearts were also compared with human heart tissue (septal myectomies from HCM patients and donor hearts as non-HCM controls).

When separated on SDS-PAGE the myofibrils of non-HCM and HCM cat hearts appeared indistinguishable from human heart myofibrils. However, when we examined the expression level of MyBP-C protein relative to α-actinin we found that five of the HCM cats had significantly reduced expression levels, compared with non-HCM cats or the other 13 HCM cats. In man it has been demonstrated that many HCM-causing mutations in *MYBPC3*, especially truncating mutations, produce a comparable haploinsufficiency of up to 25% (Marston et al., 2009, 2011; Van Dijk et al., 2009). Moreover, it has been proposed that haploinsufficiency is sufficient to cause the increased myofilament Ca^2+^-sensitivity characteristic of HCM. This result would therefore suggest that the HCM cats H6, H7, H8, H10, H14 (four outbred cats and one Bengal) and possibly H16 may have disease-causing mutations in *MYBPC3*. Unfortunately, we do not know the genotypes our cohort of HCM cats other than confirmation that the Ragdoll cat, H5, had a homozygous *MYBPC3* R820W mutation and that the Maine Coon cat, H16, did not have the *MYBPC3* A31P mutation. Other groups have likewise encountered great difficulty in genotyping cats with HCM so the question of *MYBPC3* mutations in the cats with haploinsufficiency remains unresolved at the moment (Meurs et al., 2009).

Surprisingly, the *MYBPC3* R820W mutation in H5 was not associated with any haploinsufficiency, indicating that this is not an exclusive disease-causing pathway for *MYBPC3* mutations. A recent study also failed to identify haploinsufficiency in Maine Coon cats with the A31P mutation (van Dijk et al., 2016), indicating that a poison polypeptide mechanism may be involved in HCM due to *MYBPC3* mutations in homozygous Ragdoll and Maine Coon cats. HCM not associated with MyBP-C haploinsufficiency has also been noted in some human cases with *MYBPC3* mutations (Helms et al., 2014).

Investigations of HCM in humans with known sarcomeric mutations compared with non-HCM hearts indicates that the primary abnormality induced by the mutation is likely to be enhanced Ca^2+^-sensitivity, usually accompanied by an uncoupling of the relationship between TnI phosphorylation and modulation of Ca^2+^-sensitivity (Marston, 2011, 2016). Our studies of HCM and non-HCM cat hearts likewise showed clear differences between the HCM and non-HCM samples with a 100% correspondence between HCM diagnosis and abnormality of Ca^2+^-regulation. We found that thin filaments containing HCM cat troponin always had a 1.5–2.5 fold higher Ca^2+^-sensitivity than the non-HCM containing thin filaments. This effect is as predicted for HCM-related mutations and is evidently independent of the mutated gene, which in most cases was unknown.

Interestingly, we also found that Ca^2+^-sensitivity in non-HCM cat thin filaments was modulated by TnI phosphorylation but not in any of the HCM cat samples. This “uncoupling” phenomenon, independent of the HCM-causing mutation has been noted before in human HCM and may be a common feature of HCM heart muscle even when the mutation is not in one of the troponin genes (Bayliss et al., 2012; Messer and Marston, 2014; Messer et al., 2016).

These observations of increased Ca^2+^-sensitivity of troponin together with uncoupling, found in all eight HCM cats, are surprising since mutations in troponin I, C, or T, are rare causes of HCM in human and presumably also in cats; indeed the only identified mutation is in MyBP-C. It has been reported that HCM mutations in *MYBPC3, MYH7*, and *MYL2* cause enhanced myofibrillar Ca^2+^-sensitivity in muscle strips (see Marston, 2016). This was presumed to be due to allosteric interactions between the thick and thin filaments, however the results with the cat samples indicates that troponin is itself altered secondary to the HCM mutations in another gene. It is likely that the Ca^2+^-sensitivity difference and uncoupling found in HCM troponin are related. The Ca^2+^-sensitivity of phosphorylated and unphosphorylated HCM cat troponin (EC_50_ = 0.046 ± 0.004 and 0.046 ± 0.004 μM, respectively) are equal to the Ca^2+^-sensitivity of unphosphorylated non-HCM cat troponin (EC_50_ = 0.042 ± 0.004) and are higher than that of phosphorylated non-HCM cat troponin. (EC_50_ = 0.090 ± 0.005), thus accounting for the difference in Ca^2+^-sensitivity observed when phosphorylated HCM and non-HCM cat troponin are compared (Supplementary Tables S4, S5). The apparent lack of a Ca^2+^-sensitivity difference observed with the human samples is due to the different phosphorylation levels of donor and HOCM troponin in this experiment (Figure 4B).

Experiments with troponin from human myectomy samples offer a partial explanation for these observations although further studies of human HCM samples need to be done to match those done with the cat. Supplementary Table S3 shows tests on six different human myectomy samples, five with no identified mutation and one with a *MYBPC3* mutation all of which show that the troponin Ca^2+^-sensitivity is not coupled to TnI phosphorylation level. The mechanism by which mutations not in thin filament proteins can produce uncoupling of the thin filament has not been resolved. We found that in the human myectomy samples the uncoupling was due to a secondary abnormality of TnT (Bayliss et al., 2012); despite extensive investigation, the nature of the presumed post-translational modifications of cTnT has not been found. It is, however, noteworthy that we have also been able to restore coupling in troponin from HCM cat by replacing TnT with wild-type (human) recombinant protein indicating that the same process operates in feline HCM (Supplementary Figure S4). Similarities between cat and human HCM are emphasized by the observation that the enhanced Ca^2+^-sensitivity and uncoupling due to HCM mutations can be reversed by 100 μM EGCG in the cat as has been previously demonstrated in human heart muscle (Papadaki et al., 2015).

This study shows the most established mechanism identified in humans with HCM: enhanced myofilament Ca^2+^-sensitivity and uncoupling, was present in all the feline myocardial samples tested. This finding would suggest that the disease in these cats was probably caused by mutations in myofilament proteins especially in light of the established heritability of HCM in several cat breeds (Nakagawa et al., 2002; Chetboul et al., 2012; Silverman et al., 2012; März et al., 2014). It is noteworthy that the mutations currently known only cause severe disease and reduced survival in homozygous cats (Borgeat et al., 2014; Granström et al., 2015) whilst the majority of human HCM is cases have heterozygous mutations. Whilst *MYBPC3* mutations have been identified in Maine Coon and Ragdoll cats, the causative mutation in Norwegian Forest, British Shorthair, Siberian, Sphynx, American Shorthair, Cornish Rex, Persian, Bengal, and Chartreux cats, or in any hybrid domestic shorthair cats has not been identified despite having the same disease phenotype (Meurs et al., 2009). The difficulty in determining mutations in cat genes is likely due to the sparsity of sequenced genomes. The canonical sequences of many sarcomeric proteins have not been verified by multiple independent determinations and the pattern of non-pathogenic variants is thus unknown (Montague et al., 2014).

The differences between the properties of the cat HCM samples and myectomy samples (level of TnI and MyBP-C phosphorylation and Ca^2+^-sensitivity relative to donor troponin) may relate to the different origin of the tissues sampled. The feline tissue was full thickness and obtained from the left ventricular free wall taken midway between the apex and atrioventricular groove. This area of the left ventricle is not subjected to the remodeling effect of the chronic impact of the anterior mitral leaflet during systole and associated turbulent blood flow which occurs in the basilar interventricular septum (IVS) from where myectomy samples are obtained. Significant replacement and interstitial fibrosis together with marked small intramural coronary arteriole dysplasia and myofiber disarray is common in the basal IVS for hearts with obstructive disease and may exceed that seen in the rest of the left ventricle (Fox, 2003; Moravsky et al., 2013). Alternatively, the difference may relate to the stage of disease when the samples were taken. Myectomy is performed to alleviate the left ventricular outflow tract obstruction, reduce myocardial work and thereby prolong survival (Maron and Maron, 2016). It is therefore performed in patients who still have adequate myocardial systolic function. The procedure is not available in cats and the feline samples were obtained from animals euthanized presenting with intractable heart failure, aortic thromboembolism or output failure due to advanced disease.

The study described here has strengthened the proposition that HCM in the cat is a close analog of human HCM and thus a potentially useful large animal model. The major constraint to fully exploiting the feline model of HCM is the sparsity of information about disease-causing mutations in sarcomeric and non-sarcomeric genes as described above. In addition the prevalence of HCM phenocopies and influence of modifier genes is unknown. Despite this, feline HCM has a number of significant advantages over engineered rodents as a natural proof of concept model for novel therapies due to a closer gene and protein expression profile and cardiac physiology between the cat and the human. In addition, the heterogeneous expression of the feline disease closely mimics human HCM due to the wider genetic background of cats compared to inbred mice. Finally, whilst the pathological changes characteristic of HCM may take several decades to develop in humans, the rate of progression of disease in cats generally occurs over a 2–5 year timescale enabling potential therapies to be assessed in an economically acceptable timeframe. The feline model shows promise in drug development; for instance, the effects of EGCG are essentially identical in man and cat (Papadaki et al., 2015) and the myosin inhibitor MYK-451 has been shown to exert the predicted acute physiological effect on HCM cats (Stern et al., 2016).

## Author contributions

DC and SM were joint supervisors who devised and funded the project and wrote the paper. AD, OC, and AM were responsible for the protein analysis experiments. AM and JC were responsible for the *in vitro* motility measurements. DC and AD were responsible for collecting cat heart tissue and clinical descriptions. All authors participated in data analysis and revising the paper.

### Conflict of interest statement

The authors declare that the research was conducted in the absence of any commercial or financial relationships that could be construed as a potential conflict of interest.
