# Supplementary material for: Investigations into the Sarcomeric Protein and Ca2+-Regulation Abnormalities Underlying Hypertrophic Cardiomyopathy in Cats (Felix catus)
Source: Front Physiol. 2017 Jun 8;8:348. doi: 10.3389/fphys.2017.00348 (PMC5462916; doi:10.3389/fphys.2017.00348)
Supplement: Supplementary file 5 [file DataSheet1.PDF]

Investigations into the sarcomeric protein and Ca^{2+}-regulation abnormalities underlying hypertrophic cardiomyopathy in cats (felix catus)

Andrew E Messer[1], Jasmine (Wing Shuen) Chan[2], Alex Daley[2], O'Neal Copeland[1], Steven Marston[1,3], David Connolly[2,3]

SUPPLEMENTARY DATA

1 Determination of phosphorylation levels in cat myofibrils using phosphate affinity SDS-PAGE
2 Western blots of normal and haploinsufficient cat heart samples
3 Echocardiography of cat hearts
4 The effect of exchanging in native human TnT into cat HCM sample H5 troponin.

SUPPLEMENTARY TABLES

S1 Clinical Details of the myectomy samples studied

S2 IVMA measurements comparing Ca^{2+} regulation of human myectomy (HOCM) and donor heart thin filaments

S3 IVMA measurements comparing Ca^{2+} regulation of phosphorylated and unphosphorylated human myectomy (HOCM) thin filaments

S4 P HOCM XT v uP HOCM XT (Human & Cat)

S5 P HOCM v uP HOCM ± EGCG (Human & Cat)

S6 IVMA measurements with cat heart troponin. Comparison of HCM and non-HCM and the effect of troponin phosphorylation level

SUPPLEMENTARY DATA

1 Determination of phosphorylation levels in cat myofibrils using phosphate affinity SDS-PAGE

A.

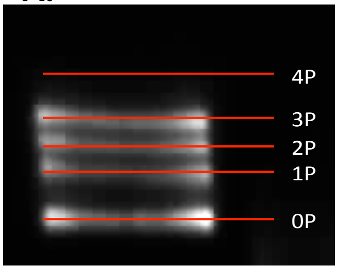

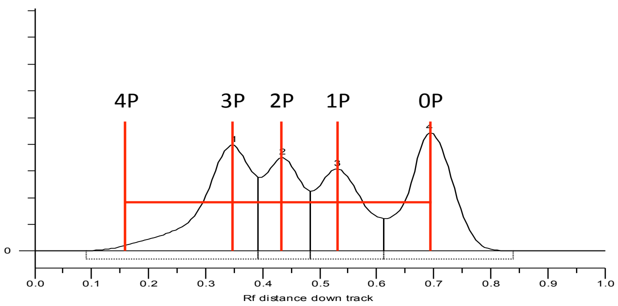

	4P	3P	2P	1P	0P	Total
Raw Volume	0	16880230	10948740	11802979	16383645	56015594
Ratio of Total Vol (RTV)	0	0.30134876	0.19545878	0.21070880	0.29248364	1
RTV x No Phosphates	0	0.90404629	0.39091757	0.21070880	0	**1.50567267**

B.

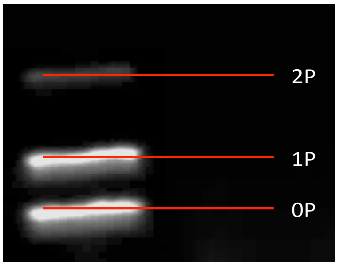

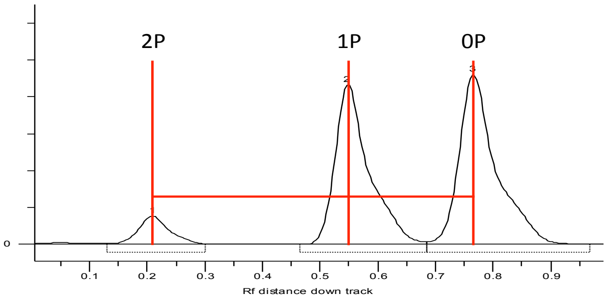

	2P	1P	0P	Total
Raw Volume	3290210.75	19953620	23097464	46341294.75
Ratio of Total Vol (RTV)	0.070999543	0.430579683	0.498420774	1
RTV x No Phosphates	0.141999086	0.430579683	0	**0.572578769**

Phosphate affinity SDS PAGE for MyBP-C and TnI with mechanism of analysis. The Phos-Tag™ added to the resolving gels binds PO_4^{2-} groups on proteins. This increases the protein's mass in a stepwise fashion dependant on number of PO_4^{2-} groups attached. When run on SDS PAGE proteins are separated into phosphorylation levels. **A)** Image showing the 5 phosphorylation levels of MyBP-C (4P band present in other samples). Densitometry was used to measure the volume of each band (each relating to a phosphorylation level) and the ratio of total MyBP-C at that phosphorylation level was found. Total moles PO_4^{2-}/mole MyBP-C (a measure of phosphorylation of MyBP-C in the whole sample) was found by multiplying those ratios by the number of phosphates bound at that level, then adding those values together (0 x ratio P0 + 1 x ratio P1 … + 4 x ratio P4). Shown in bold in bottom-right of table. **B)** Image showing the 3 phosphorylation levels of TnI (meaning it has 2 PO_4^{2-} binding sites). Moles phosphate/Mole TnI was calculated in the same way.

2 Western blots of normal and haploinsufficient cat heart samples.

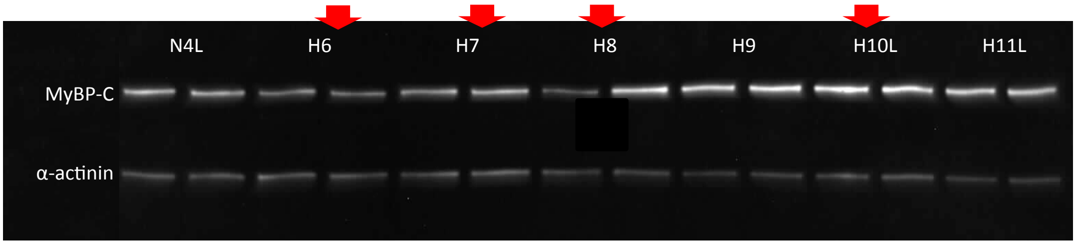

Western blot of feline cardiac samples labelled with antibodies specific to MyBP-C N-terminus and to α-actinin as a loading control. Duplicate lanes for each sample. The samples exhibiting haploinsufficiency are indicated with arrows; there is no evidence of truncated peptide of MyBP-C.

3 Echocardiography of cat hearts

See also Figure 1 e-h and supplementary movies

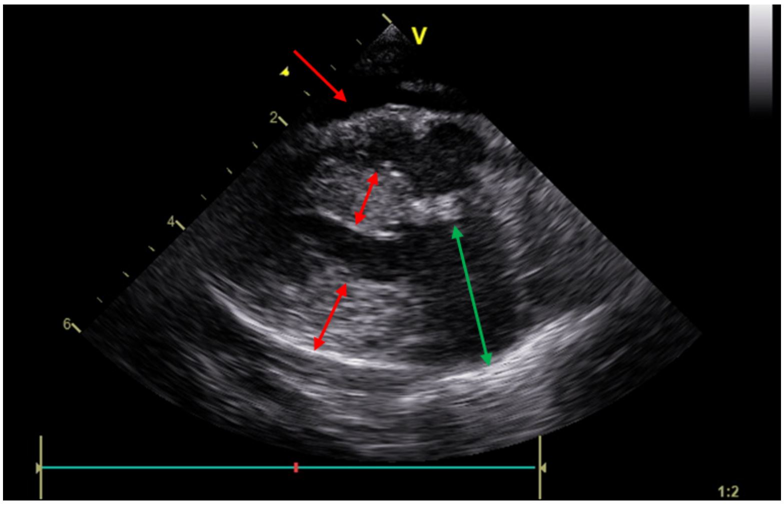

Right parasternal long axis view of cat H12 from an emergency cage-side echo. Although the image quality is reduced, the phenotypic changes are clearly visible. Note enlarged LA (green arrow) and severe hypertrophy of the LV free wall and interventricular septum measuring > 6mm (red double headed arrows). A small volume pericardial effusion (red single headed arrow) secondary to heart failure is also present (see accompanying movie S3)

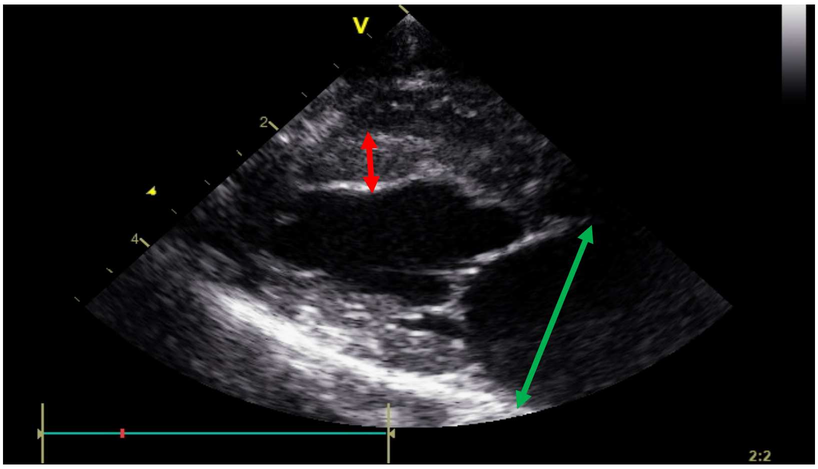

Right parasternal long axis view of cat H5 (Ragdoll homozygous for the MYBPC3 R820W mutation) at end diastole. Note the thickened interventricular septum measuring 6.2mm (red arrow) and the enlarged LA (green arrow). See accompanying movie S4.

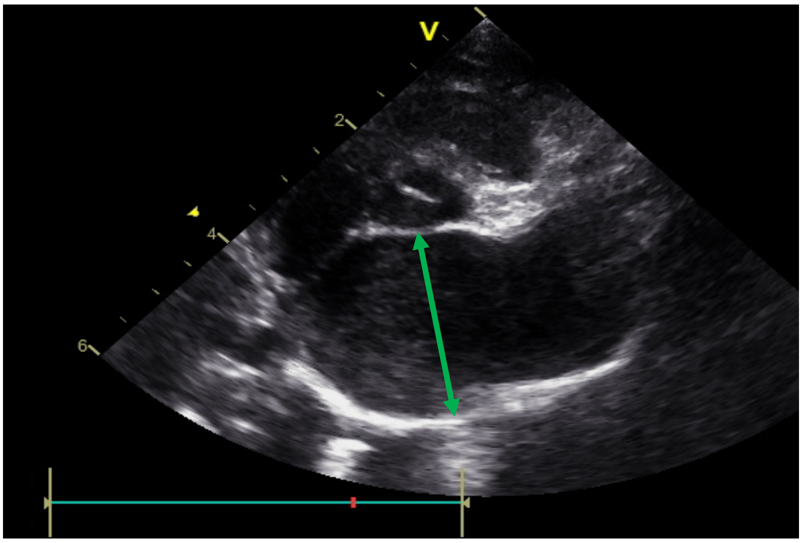

Right parasternal short axis view at the heart base of cat H5 at end systole. Note the significantly enlarged LA (green arrow). See accompanying movie S4

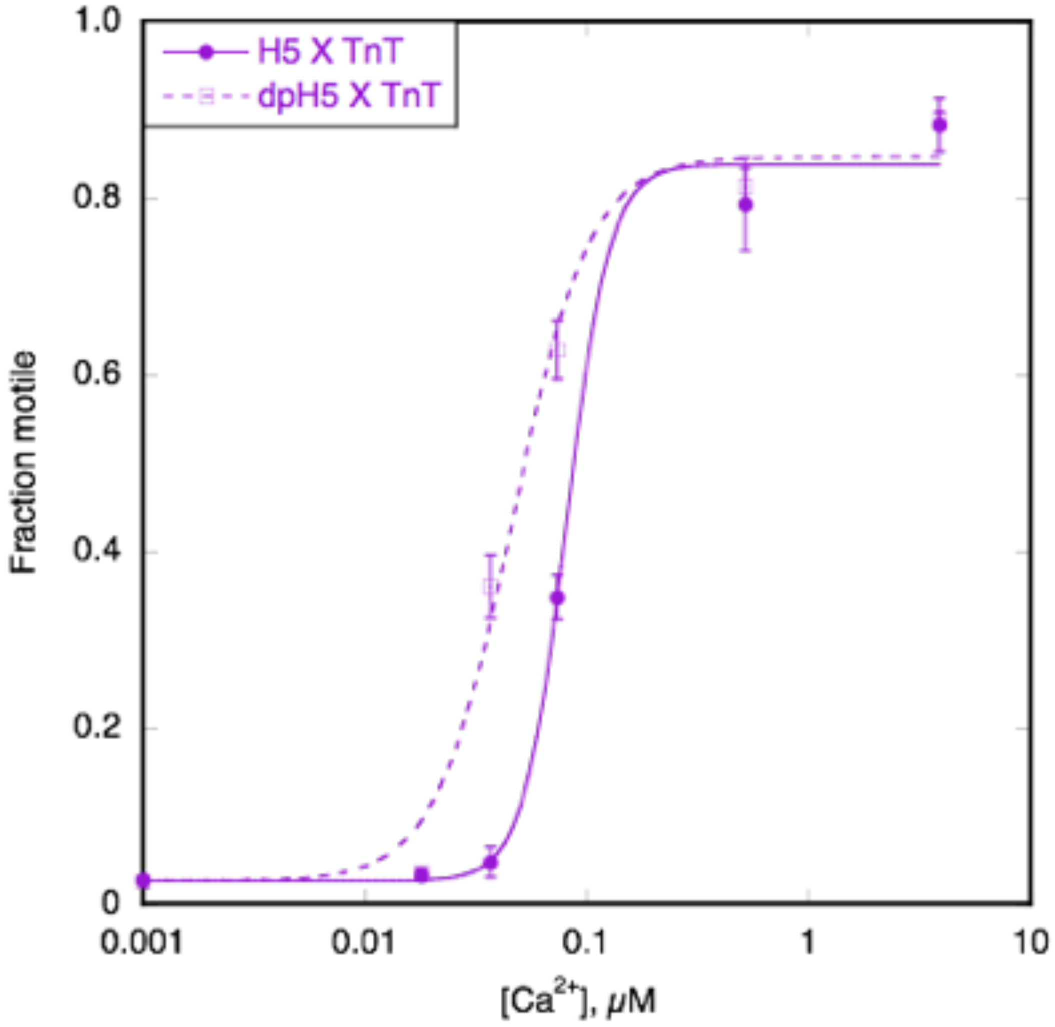

S4 The effect of exchanging in native human TnT into cat HCM sample H5. The Hill equation is fitted to the data to yield values of EC_{50} and n_H. Solid symbols and line, phosphorylated; open symbols and dotted line, unphosphorylated. Coupling is restored, compare with Figure 6. Full data in Supplementary Table S4.

.

.

Table S1 Patient Clinical details

Biopsy sample	M1	M3	M4	M5	M15	MD	ML	MM	MP	MR	MT	MV	MH1	3.166
**Mutation**	*MYH7* R719Q	NO MUTATION FOUND MYH7, MYBPC3, TNNT2, TNNT3, TPM1	NO MUTATION FOUND MYH7, MYBPC3, TNNT2, TNNT3, TPM1	*MYH7* V606M	*MYBPC* T2604A+, ΔC2605	NO MUTATION FOUND MYH7, MYBPC3, TNNT2, TNNT3, TPM1	*MYBPC3* IVS17+4A>T	NO MUTATION FOUND MYH7, MYBPC3, TNNT2, TNNT3, TPM1	NO MUTATION FOUND MYH7, MYBPC3, TNNT2, TNNT3, TPM1	NO MUTATION FOUND MYH7, MYBPC3, TNNT2, TNNT3, TPM1	*MYBPC3* IVS17+4A>T	NO MUTATION FOUND MYH7, MYBPC3, TNNT2, TNNT3, TPM1	*MYBPC3* 1624G>C (E542Q)	*TNNT2* K280N (homozygous)
**Diagnosis**	HCM	HCM	HCM	HCM	HCM	HCM	HCM	HCM	HCM	HCM	HCM	HCM	HCM	HCM
**Age**	24	42	49	31	42	48	46	58	62	33	46	53	34	26
**Gender**	F	M	M	M	M	M	F	M	F	M	F	M	M	M
**Family History**	No HCM No SCD	No HCM No SCD	Father died 58yrs ? cause	No HCM, No SCD, 9 siblings –ve	Father died 49yrs ? SCD								Father had SCD	Father has CAD and angina
**Current Treatment**	Verapamil Amiodarone	Atenolol Diso-pyramide	Atenolol Aspirin Diso-pyramide Clopidogrel Quinine	Propanolol	Verapamil Disopyramide Warfarin Salbutamol									
**ECHO appearance**	ASH	ASH	Concentric LVH	Mild ASH	ASH									
**MaxLVWT (mm)**	27.5	23	16	20	23			12					30	
**Max. ST(mm)**	27.5	23	16	20	23			19			11			
**LVEDD (mm)**		43.6	42.0	39.0	46		45	50			16			
**LVESD (mm)**		23.9	28.0	21.0	30		25	31			45			
**LA (mm)**	45	41	48	41	49		41	46			25			
**FS (%)**		45	33	48	35		44	EF 68%			41			
**SAM**	Complete	Incomplete	Complete	Complete	incomplete		Complete	complete			44			
**MR**	Mild	Mild	Mild to Mod.	Trivial	Mild		Mild	moderate			complete			
**Resting LVOT Gradient**	86mmHg	22mmHg (133mmHg with GTN)	104mmHg	110mmHg	44mmHg (Valsalva 116mmHg)		110	103			mild		120	
**VT on Holter Monitor**	VT (non-sustained)	No	Isolated VE's only	No	No		No VT	No			110mmHg			
**ETT MVO2 (ml/min/kg)**	58% predicted No arrhythmias Flat BP	20.3 68% predicted No arrhythmias BP rise	11.7 35% predicted No arrhythmias Flat BP	62% predicted No arrhythmia Flat BP	28.1 78% predicted No arrhythmias BP rise			68% predicted			no			
**ECG**	SR, LVH,RA	SR, LVH, RA	SR, LVH	SR, LVH	Paced			SR LBBB						
**NYHA Class**	II	II-III	II	III	III						III		II/III	IV

Table S2 human HOCM v Donor

Sample	EC_{50} sliding speed, μM		EC_{50} ratio HOCM/ Donor	Hill co-efficient		Max speed at 3.9μM Ca^{2+}	
	HOCM	Donor		HOCM	Donor	HOCM	Donor
M1	0.18±0.06	0.19±0.05	0.95	1.06	1.09	3.59	3.64
M2	0.20±0.04	0.19±0.04	1.04	1.46	1.48	3.99	3.94
M3	0.10±0.02	0.12±0.02	0.84	1.57	1.40	3.36	3.39
M4	0.12±0.02	0.12±0.02	0.97	1.74	1.64	4.21	4.14
M4	0.13±0.02	0.16±0.02	0.77	1.56	1.36	4.50	4.47
M15	0.25±0.03	0.36±0.18	0.69	1.55	1.46	3.86	3.92
Mean	0.16±0.02	0.19±0.04	0.88±0.05	1.49± 0.09	1.41± 0.07	3.92± 0.17	3.92± 0.15
p	0.19/0.55		0.07				

Sample	EC_{50} fraction motile, μM		EC_{50} ratio HOCM/ Donor	Hill co-efficient		Max fraction motile at 3.9μM Ca^{2+}	
	HOCM	Donor		HOCM	Donor	HOCM	Donor
M1	0.05±0.003	0.05±0.004	0.94	3.93	4.24	0.88	0.87
M2	0.05±0.003	0.05±0.003	1.04	2.62	2.34	0.82	0.83
M3	0.07±0.004	0.07±0.004	0.96	2.06	1.74	0.90	0.88
M4	0.10±0.02	0.09±0.01	1.03	1.50	1.52	0.86	0.87
M4	0.07±0.01	0.08±0.01	0.87	2.56	1.91	0.97	0.98
M15	0.12±0.07	0.16±0.13	0.75	0.80	0.70	0.72	0.74
MT	0.10±0.51	0.10±0.02	0.92	4.65	2.12	0.69	0.73
MT	0.13±0.11	0.19±0.04	0.66	1.22	1.64	0.70	0.76
MT	0.16±0.02	0.21±0.04	0.73	2.18	1.85	0.79	0.79
MT	0.14±0.01	0.18±0.09	0.74	3.93	1.69	0.72	0.72
MT	0.11±0.06	0.10±0.01	1.09	4.90	5.97	0.83	0.87
MT	0.23±0.05	0.36±0.16	0.63	2.73	1.13	0.85	0.82
MT	0.21±0.05	0.18±0.04	1.15	1.95	2.42	0.74	0.66
MT	0.23±0.06	0.21±0.01	1.11	4.56	2.79	0.74	0.78
Mean	0.13±0.02	0.15±0.02	0.90±0.05	2.83± 0.36	2.29± 0.36	0.80± 0.02	0.81± 0.02
p	0.12/0.51		0.05				

Table S3 Human P HOCM v uP HOCM

Sample	EC_{50} sliding speed, µM		EC_{50} ratio P HOCM/ uP HOCM	Hill co-efficient		Max speed at 3.9µM Ca^{2+}	
	P HOCM	uP HOCM		P HOCM	uP HOCM	P HOCM	uP HOCM
MR	0.046±0.004	0.048±0.006	0.96	2.79	2.83	3.34	3.46
MR	0.047±0.004	0.050±0.006	0.94	2.26	2.33	3.14	3.23
MH1	0.071±0.099	0.072±0.003	0.99	6.91	7.98	2.86	2.99
MH1	0.076±0.001	0.074±0.130	1.03	4.67	9.52	3.20	3.07
MH1	0.064±0.008	0.063±0.002	1.02	5.98	5.82	2.48	2.26
MD	0.092±0.006	0.102±0.008	0.90	4.67	4.65	2.97	2.88
MD	0.075±0.007	0.057±0.002	1.32	4.05	7.92	2.78	2.85
MD	0.068±0.007	0.063±0.004	1.08	4.13	4.95	3.08	2.96
MV	0.086±0.008	0.105±0.012	0.82	3.32	2.91	3.09	3.20
MV	0.123±0.021	0.120±0.035	1.03	4.65	3.40	3.36	3.36
MV	0.089±0.004	0.096±0.001	0.93	8.21	9.43	2.94	2.82
MV	0.042±0.008	0.045±0.009	0.93	1.97	1.88	3.02	3.11
MV	0.041±0.009	0.042±0.009	0.98	1.94	1.99	2.91	2.90
MV	0.052±0.014	0.048±0.011	1.08	1.64	1.79	3.05	3.04
MD	0.057±0.009	0.052±0.008	1.09	1.74	1.82	3.13	3.06
MD	0.050±0.007	0.052±0.009	0.96	1.72	1.75	3.07	3.09
MD	0.055±0.015	0.055±0.014	1.00	1.43	1.49	3.12	3.11
Mean	0.067±0.005	0.067±0.006	1.00± 0.03	3.65± 0.49	4.26± 0.69	3.03± 0.05	3.02± 0.06
p	0.76/0.94		0.89				

Table S3 cont.

Sample	EC_{50} fraction motile, µM		EC_{50} ratio P HOCM/ uP HOCM	Hill co-efficient		Max fraction motile at 3.9µM Ca^{2+}	
	P HOCM	uP HOCM		P HOCM	uP HOCM	P HOCM	uP HOCM
MM	0.17±0.11	0.23±0.06	0.74	0.95	2.43	0.78	0.78
MM	0.12±0.05	0.09±0.02	1.33	1.28	1.38	0.75	0.81
MM	0.16±0.14	0.14±0.08	1.14	0.65	1.35	0.80	0.81
MM	0.15±0.02	0.13±0.01	1.15	1.81	5.88	0.76	0.72
MM	0.12±0.02	0.14±0.09	0.86	2.25	2.87	0.75	0.73
MM	0.24±0.06	0.27±0.06	0.89	1.24	1.31	0.71	0.74
MM	0.09±0.01	0.08±0.02	1.13	4.41	2.18	0.76	0.74
MM	0.11±0.02	0.17±0.02	0.65	2.43	2.63	0.72	0.75
MR	0.042±0.003	0.043±0.005	0.98	2.77	2.43	0.80	0.83
MR	0.039±0.0001	0.040±0.001	0.98	2.66	2.37	0.79	0.80
MH1	0.033±0.004	0.034±0.008	0.98	3.99	2.88	0.74	0.84
MH1	0.057±0.0004	0.060±0.003	0.95	3.88	4.63	0.81	0.81
MH1	0.060±0.006	0.069±0.007	0.87	4.95	6.83	0.71	0.72
MD	0.069±0.004	0.062±0.001	1.11	7.80	5.07	0.91	0.88
MD	0.042±0.005	0.043±0.0002	0.98	2.10	7.64	0.89	0.93
MD	0.058±0.002	0.057±0.002	1.02	5.71	5.59	0.92	0.91
MV	0.058±0.004	0.053±0.009	1.09	8.83	13.32	0.87	0.80
MV	0.063±0.008	0.064±0.002	0.98	8.62	7.19	0.88	0.85
MV	0.055±0.008	0.048±0.004	1.15	4.19	13.71	0.85	0.81
MV	0.048±0.004	0.049±0.003	0.98	2.32	2.30	0.86	0.88
MV	0.050±0.003	0.051±0.004	0.98	2.54	2.31	0.83	0.87
MV	0.061±0.006	0.060±0.005	1.02	2.05	2.17	0.87	0.87
MD	0.055±0.003	0.054±0.004	1.02	2.54	2.71	0.84	0.83
MD	0.055±0.004	0.052±0.004	1.06	2.48	2.66	0.82	0.82
MD	0.053±0.006	0.053±0.004	1.00	2.58	2.67	0.85	0.82
Mean	0.082±0.010	0.086±0.012	1.00± 0.03	3.40± 0.45	4.26± 0.67	0.81± 0.01	0.81± 0.01
p	0.42/0.83		0.95				

Table S4 P HOCM XT v uP HOCM XT in human and cat

Sample	EC$_{50}$ sliding speed, µM		EC$_{50}$ ratio P HOCM XT/uP HOCM XT	Hill co-efficient		Max speed at 3.9µM Ca^{2+}	
	P HOCM XT	uP HOCM XT		P HOCM XT	uP HOCM XT	P HOCM XT	uP HOCM XT
HUMAN							
ML	0.17±0.01	0.10±0.01	1.73	1.68	1.58	2.80	2.74
3.166	0.27±0.03	0.12±0.01	2.23	1.34	1.07	2.71	2.63
3.166	0.13±0.03	0.09±0.01	1.42	1.38	1.70	2.85	2.93
MP	0.13±0.03	0.07±0.01	1.91	1.35	1.42	2.39	2.40
MP	0.10±0.02	0.05±0.01	1.96	1.17	1.17	2.59	2.60
Mean	0.16±0.03	0.086±0.01	1.85±0.13	1.38± 0.08	1.39± 0.12	2.67± 0.08	2.66± 0.09
p	0.020/0.050		0.0032				
CAT							
H5	0.080±0.003	0.047±0.005	1.70	3.60	2.38	3.06	3.09
H5	0.078±0.001	0.045±0.005	1.73	4.59	2.14	2.96	3.14
H5	0.077±0.004	0.048±0.005	1.71	4.71	2.07	3.10	3.02
Mean	0.078±0.001	0.047±0.001	1.71±0.01	4.30± 0.35	2.20± 0.09	3.04± 0.04	3.08± 0.03
p	0.018/<0.0001		0.0002				

Sample	EC$_{50}$ fraction motile, µM		EC$_{50}$ ratio P HOCM XT/uP HOCM XT	Hill co-efficient		Max fraction motile at 3.9µM Ca^{2+}	
	P HOCM XT	uP HOCM XT		P HOCM XT	uP HOCM XT	P HOCM XT	uP HOCM XT
HUMAN							
ML	0.14±0.02	0.06±0.002	2.29	1.85	1.61	0.97	0.97
3.166	0.13±0.02	0.07±0.004	1.91	2.05	2.05	0.95	0.95
3.166	0.10±0.004	0.07±0.002	1.52	3.33	2.56	0.93	0.96
MP	0.17±0.03	0.10±0.01	1.71	1.66	2.07	0.94	0.95
MP	0.15±0.02	0.09±0.001	1.69	1.93	2.05	0.93	0.93
Mean	0.14±0.01	0.078±0.01	1.82±0.13	2.16± 0.30	2.07± 0.15	0.94± 0.01	0.95± 0.01
p	0.0020/0.0024		0.0033				
CAT							
H5	0.081±0.001	0.051±0.003	1.59	4.57	2.52	0.87	0.87
H5	0.081±0.006	0.047±0.005	1.72	4.50	2.53	0.88	0.89
H5	0.080±0.004	0.050±0.004	1.60	4.44	2.39	0.89	0.88
Mean	0.081±0.0003	0.049±0.001	1.64±0.04	4.50± 0.04	2.48± 0.05	0.88± 0.01	0.88± 0.01
p	0.0018/<0.0001		0.0043				

Table S5 P HOCM v uP HOCM ± EGCG in human and cat

Sample	EC_{50} sliding speed, µM (- EGCG)		EC_{50} ratio P HOCM/ uP HOCM	EC_{50} sliding speed, µM (+ EGCG)		EC_{50} ratio P HOCM/ uP HOCM + EGCG
	P HOCM	uP HOCM		P HOCM	uP HOCM	
HUMAN						
MR	0.046±0.004	0.048±0.006	0.96	0.068±0.013	0.045±0.004	1.51
MR	0.047±0.004	0.050±0.006	0.94	0.089±0.016	0.051±0.004	1.75
MV	0.042±0.008	0.045±0.009	0.93	0.089±0.024	0.038±0.006	2.34
MV	0.041±0.009	0.042±0.009	0.98	0.109±0.023	0.042±0.009	2.60
MV	0.052±0.014	0.048±0.011	1.08	0.114±0.010	0.051±0.011	2.24
MD	0.057±0.009	0.052±0.008	1.09	0.076±0.003	0.041±0.005	1.85
MD	0.050±0.007	0.052±0.009	0.96	0.077±0.007	0.043±0.005	1.79
MD	0.055±0.015	0.055±0.014	1.00	0.097±0.020	0.043±0.007	2.26
Mean	0.049±0.002	0.049±0.001	0.99± 0.02	0.090±0.006	0.044±0.002	2.04± 0.13
p	0.83/0.92		0.74	<0.0001/<0.0001		<0.0001
CAT						
H13	0.062±0.015	0.063±0.009	0.99	0.189±0.03	0.056±0.001	3.38
H14	0.066±0.013	0.059±0.009	1.12	0.056±0.011	0.040±0.003	1.40
H5	0.045±0.009	0.047±0.008	0.96	0.097±0.009	0.047±0.006	2.06
H5	0.052±0.009	0.053±0.007	0.99	0.081±0.009	0.045±0.007	1.82
H5	0.054±0.008	0.052±0.009	1.04	0.116±0.029	0.046±0.006	2.52
H5	0.044±0.008	0.046±0.009	0.96	0.097±0.015	0.040±0.005	2.43
Mean	0.054±0.004	0.053±0.003	1.01± 0.03	0.106±0.020	0.046±0.002	2.27± 0.28
p	0.74/0.91		0.71	0.014/0.0090		0.006

Sample	EC50 fraction motile, μM (- EGCG)		EC50 ratio P HOCM/ uP HOCM	EC50 fraction motile, μM (+ EGCG)		EC50 ratio P HOCM/ uP HOCM + EGCG
	P HOCM	uP HOCM		P HOCM	uP HOCM	
HUMAN						
MR	0.042±0.003	0.043±0.005	0.98	0.070±0.003	0.044±0.001	1.59
MR	0.039±0.0001	0.040±0.001	0.98	0.076±0.006	0.042±0.006	1.81
MV	0.048±0.004	0.049±0.003	0.98	0.109±0.029	0.048±0.005	2.27
MV	0.050±0.003	0.051±0.004	0.98	0.100±0.006	0.053±0.005	1.89
MV	0.061±0.006	0.060±0.005	1.02	0.126±0.022	0.063±0.009	2.02
MD	0.055±0.003	0.054±0.004	1.02	0.088±0.020	0.040±0.005	2.22
MD	0.055±0.004	0.052±0.004	1.06	0.112±0.044	0.040±0.005	2.80
MD	0.053±0.006	0.053±0.004	1.00	0.133±0.027	0.044±0.005	3.02
Mean	0.050±0.003	0.050±0.002	1.00± 0.01	0.102±0.008	0.047±0.003	2.20± 0.17
p	0.82/0.97		0.82	0.0001/<0.0001		0.0002
CAT						
H13	0.030±0.004	0.029±0.006	1.03	0.068±0.006	0.035±0.005	1.94
H14	0.039±0.008	0.046±0.009	0.85	0.048±0.006	0.037±0.001	1.30
H5	0.047±0.004	0.048±0.006	0.98	0.086±0.003	0.053±0.005	1.62
H5	0.050±0.006	0.048±0.005	1.04	0.090±0.001	0.056±0.006	1.61
H5	0.057±0.008	0.056±0.007	1.02	0.104±0.008	0.059±0.008	1.76
H5	0.046±0.005	0.050±0.006	0.92	0.130±0.016	0.051±0.007	2.55
Mean	0.045±0.004	0.046±0.004	0.97± 0.03	0.088±0.012	0.049±0.004	1.80± 0.17
p	0.39/0.81		0.42	0.008/0.010		0.006

Supplementary Table 6: All Cat data

Velocity | Percentage | N.A.

	DATE	WT	sd	n_H	Mutant	sd	n_H	WT / H EC50	DATE	P	sd	n_H	dP	sd	n_H	P/dP EC50
N4									14.04.16B	0.078425	0.01467	2.446	0.050351	0.00257	4.6442	1.557565887
									14.04.16C	0.099431	0.01242	1.9283	0.050682	0.0028	3.6046	1.961860227
									14.04.16B	0.060272	0.01171	8.4752	0.032587	0.00295	6.5148	1.849571915
									14.04.16C	0.062093	0.00686	7.191	0.034901	0.00261	6.1738	1.779118077
N7									27.04.16A	0.068707	0.00351	3.74	0.03731	0.00486	2.2638	1.84151702
									28.04.16C	0.061377	0.00504	5.065	0.026602	0.00402	6.169	2.307232539
									27.04.16A	0.058397	0.00806	5.7217	0.030241	0.00222	3.3596	1.931053867
									28.04.16C	0.054886	0.00451	5.5735	0.030201	0.00276	4.9017	1.817357041
N8									06.04.16A	0.076684	0.00688	3.6719	0.05734	0.00489	2.303	1.337356121
									06.04.16A	0.061887	0.01048	6.8075	0.043226	0.00534	3.7562	1.431707768
									06.04.16B	0.054981	0.00974	7.8756	0.032772	0.00419	4.7016	1.677682168
N11 (Mai Tai)									05.05.16A	0.073075	0.00352	3.0961	0.034285	0.00146	3.4079	2.131398571
									05.05.16B	0.065452	0.00114	3.3759	0.034274	0.00195	5.2913	1.909669137
									05.05.16A	0.061275	0.00246	5.2644	0.030858	0.00221	2.4697	1.98570873
									05.05.16B	0.062593	0.00628	5.9922	0.035429	0.00304	2.976	1.766716532
N12 (Miggy)									11.05.16A	0.075667	0.00997	2.6901	0.046655	0.00952	2.2893	1.621841175
									11.05.16A	0.05774	0.00573	5.6041	0.030023	0.00295	3.8183	1.923192219
H1									16.03.16	0.04962	0.00586	5.2882	0.041036	0.00459	19.01	1.209182181
									17.03.16A	0.070067	0.03632	16.115	0.072051	0.01545	15.97	0.972463949
									16.03.16	0.032955	0.00366	4.256	0.035562	0.00481	7.5117	0.926691412
									17.03.16A	0.046961	0.01142	2.9873	0.043239	0.00694	6.5877	1.086079697
H2									17.02.16	0.042141	0.00774	1.9622	0.039416	0.00362	2.3773	1.069134362
									18.02.16A	0.04982	0.00125	2.598	0.050151	0.0038	2.2105	0.993399932
									18.02.16B	0.03731	0.00268	4.1022	0.041427	0.00198	3.5843	0.900620368
									17.02.16	0.044297	0.00958	2.3756	0.041518	0.01222	1.8535	1.066934823
									18.02.16A	0.03643	0.01094	2.3224	0.040404	0.01526	1.9469	0.901643402
									18.02.16B	0.037219	0.00744	2.4824	0.040903	0.00491	2.1895	0.909933257
H3	27.07.16A	0.19636	0.0178	1.3236	0.066622	0.0049	3.9161	2.947374741	28.07.16A	0.077628	0.01292	2.1622	0.085047	0.01988	2.2963	0.912765882
and (vs N12)	27.07.16B	0.11069	0.0371	3.9552	0.06182	0.0022	5.0049	1.790520867	28.07.16B	0.14272	0.01912	1.5505	0.11738	0.02608	2.1709	1.215880048
	27.07.16C	0.1097	0.0144	2.6622	0.075981	0.0042	4.0428	1.443781998	28.07.16C	0.071448	0.01142	2.9564	0.066408	0.00072	3.1296	1.075894471
	27.07.16A	0.070616	0.0547	20.4444	0.039115	0.0013	6.7115	1.805343219	28.07.16A	0.03584	0.00081	4.4868	0.035052	0.00131	4.3949	1.022480886
	27.07.16B	0.089478	0.0035	2.7748	0.038367	0.0015	4.0029	2.33216045	28.07.16B	0.03378	0.00285	3.6028	0.034757	0.0033	3.2367	0.971890554
	27.07.16C	0.056475	0.0017	7.4443	0.038605	0.0009	4.521	1.462893408	28.07.16C	0.033089	0.00101	4.7	0.032778	0.00236	6.1342	1.009488071
H5									13.05.15A	0.042803	0.01143	1.243	0.036736	0.00948	1.4606	1.16515135
									13.05.15B	0.062491	0.01963	1.1007	0.053688	0.01217	1.2246	1.163965877
									14.05.15	0.069302	0.01565	1.1914	0.059585	0.01518	1.3467	1.163077956
									03.11.16B	0.044743	0.0086	1.8602	0.046572	0.00777	1.9146	0.960727476
									16.11.16A	0.052456	0.00935	1.7225	0.052566	0.00711	1.802	0.997907393
									16.11.16B	0.054186	0.00767	1.8048	0.051975	0.00895	1.7775	1.042539683
									17.11.16	0.044211	0.00764	1.9954	0.045686	0.00893	1.9708	0.967714398
									13.05.15A	0.051625	0.01123	1.4319	0.039648	0.00838	1.5424	1.302083333
									13.05.15B	0.05102	0.01194	1.4542	0.042811	0.00947	1.4924	1.191749784
									14.05.15	0.075451	0.01799	1.2484	0.053339	0.01003	1.5788	1.414555953
									03.11.16B	0.04744	0.00432	2.3789	0.047875	0.00583	2.2313	0.990913838
									16.11.16A	0.049525	0.00592	2.0691	0.048446	0.00502	2.1984	1.022272221
									16.11.16B	0.056605	0.00813	1.8969	0.056137	0.00712	1.9474	1.008336748
									17.11.16	0.046389	0.00518	2.279	0.050089	0.00598	2.1249	0.926131486
H5	06.05.15A	0.09325	0.0024	1.9277	0.042403	0.0081	1.0217	2.199136854								
and (vs N4)	06.05.15B	0.10568	0.0087	1.8785	0.048964	0.0129	1.0733	2.158320399								
	07.05.15	0.12736	0.0399	1.1003	0.023728	0.012	0.60936	5.367498314								
	06.05.15A	0.091922	0.007	2.5034	0.03154	0.0073	1.5975	2.914457831								
	06.05.15B	0.10647	0.0044	1.9017	0.038715	0.008	1.4192	2.750096862								
	07.05.15	0.10667	0.0025	2.2523	0.039261	0.0058	1.2489	2.716945569								
H5	04.02.16A	0.11115	0.0649	3.3572	0.058956	0.0108	2.3929	1.885304295	10.02.16A	0.062234	0.00215	2.3296	0.053215	0.00121	2.5327	1.169482289
and (vs N7)	04.02.16B	0.096387	0.0401	3.8657	0.067365	0.0061	2.7147	1.43081719	10.02.16B	0.058192	0.00498	2.0851	0.053879	0.00817	2.49	1.080049741
	05.02.16	0.10324	0.0147	2.3461	0.057652	0.0102	2.4963	1.790744467	11.02.16	0.087428	0.01575	1.5368	0.068694	0.01474	1.8288	1.272716686
	04.02.16A	0.080066	0.0087	5.011	0.051567	0.0112	3.323	1.552659647	10.02.16A	0.04233	0.01681	2.0787	0.036726	0.00547	2.5885	1.152589446
	04.02.16B	0.079261	0.0036	5.1315	0.058397	0.0061	2.9383	1.357278627	10.02.16B	0.036591	0.00489	2.721	0.033631	0.00602	3.0369	1.088014035
	05.02.16	0.09876	0.0159	1.8611	0.052436	0.0068	2.8262	1.883438859	11.02.16	0.045278	0.00482	1.9568	0.043808	0.00547	2.045	1.033555515
H7	27.04.16C	0.06275	0.0093	1.9019	0.034256	0.0134	1.6794	1.83179589	28.04.16A	0.039544	0.010991	1.9065	0.042233	0.005345	2.1564	0.936329411
and (vs N7)									28.04.16B	0.036783	0.016721	1.7535	0.03759	0.01214	1.8668	0.978531524
	27.04.16B	0.058652	0.0037	4.0602	0.026402	0.0039	5.4974	2.221498371	28.04.16A	0.029264	0.002801	3.6728	0.029188	0.001992	4.5991	1.00260381
	27.04.16C	0.053818	0.0021	4.0659	0.026759	0.0038	5.1348	2.011211181	28.04.16B	0.032227	0.00591	2.1543	0.029317	0.00387	3.9515	1.099259815
H12	13.04.16A	0.078933	0.0089	2.4381	0.057861	0.0017	4.1312	1.364183129	13.04.16C	0.04761	0.00846	2.1049	0.042432	0.0053	1.8851	1.122030543
and (vs N4)	13.04.16B	0.064737	0.0102	5.087	0.053933	0.0032	5.0164	1.200322623	14.04.16A	0.055362	0.0136	3.9404	0.061598	0.01028	2.6985	0.898762947
	13.04.16A	0.047642	0.0072	7.963	0.035606	0.0008	4.5198	1.338032916	13.04.16C	0.029041	0.00179	4.3956	0.030351	0.00211	5.6692	0.956838325
	13.04.16B	0.04901	0.0092	8.9231	0.031325	0.0005	5.6065	1.564565044	14.04.16A	0.03421	0.00388	5.4395	0.037446	0.00471	3.0007	0.913582225
H13	11.05.16B	0.07934	0.0087	3.5097	0.033428	0.0087	2.634	2.373459375	12.05.16C	0.062676	0.01526	1.5583	0.063271	0.00902	1.8502	0.990596008
and (vs N12)	11.05.16B	0.053072	0.0034	5.9333	0.029514	0.0014	4.625	1.798197466	12.05.16A	0.029924	0.00368	5.99	0.028897	0.00556	5.6913	1.035540021
									12.05.16C	0.023868	0.00292	5.6663	0.022501	0.00257	5.6576	1.060752855
H14	20.05.15A	0.10677	0.0102	0.76144	0.039899	0.0192	1.7497	2.676006917	04.05.16A	0.065856	0.01282	1.2876	0.058848	0.00897	3.0908	1.11908646
and (vs N7)	20.05.15B	0.083432	0.0149	1.1294	0.040975	0.0137	1.6086	2.036168395	04.05.16B	0.08856	0.01926	0.91495	0.10112	0.03033	0.84673	0.875791139
	21.05.15	0.080887	0.0276	1.0813	0.03222	0.0053	4.2535	2.510459342								
	20.05.15A	0.092131	0.0247	1.1909	0.050826	0.0124	1.7969	1.812674615	04.05.16A	0.038574	0.00842	1.6738	0.045893	0.00917	1.8623	0.840520341
	20.05.15B	0.10044	0.0178	1.3602	0.065805	0.0172	1.3086	1.526327787	04.05.16B	0.032814	0.01163	1.5645	0.039252	0.01177	1.7071	0.83598288
	21.05.15	0.11587	0.0212	1.3016	0.076837	0.0245	1.2969	1.507997449								

S6 IVMA measurements with cat heart troponin. Comparison of HCM and non-HCM and the effect of troponin phosphorylation level
